# Chitosan–graphene oxide films as a promising packaging material for the preservation of fresh plums (*Prunus salicina* Lindl.)

**DOI:** 10.1039/d5ra05155j

**Published:** 2026-01-06

**Authors:** Tran Y. Doan Trang, Phan Khiet Suong, Duong Van Thiet, Nguyen Quang Tung, Ha Thi-Dung, Vu Phuong Lan, Nguyen The Huu, Do Thi Hanh

**Affiliations:** a Institute of Technology – HaUI, Hanoi University of Industry Vietnam tydtrang@gmail.com tydtrang@haui.edu.vn; b Faculty of Chemical Technology, Hanoi University of Industry Vietnam; c School of Mechanical Engineering and Automotive Technology, Hanoi University of Industry Vietnam

## Abstract

This study investigated the properties of bio-based films composed of chitosan (CH) at concentrations of 1.25% and 1.5%, incorporated with various amounts of graphene oxide (GO) (0, 0.5, 1.0, 1.5, and 2.0%), to develop films for fresh plum preservation. The results showed that films containing low concentrations of GO (0.5–1.0%) exhibited high transparency, whereas higher GO content (1.0–2.0%) led to a significant reduction in transparency. The addition of GO increased the surface roughness of the films. It caused noticeable color changes: brightness decreased, the characteristic reddish-yellow hue of pure chitosan was lost, resulting in darker and less vibrant films. Furthermore, the CH–GO films demonstrated relatively good near-UV blocking ability, allowing only visible light in the yellow to red spectrum to pass through. The incorporation of GO slightly reduced the water absorption and solubility, while significantly decreasing the water vapor permeability of the material (by 47.09–50.58% compared to the control film). In terms of mechanical properties, the film containing a low GO concentration (1.0%) exhibited a markedly higher tensile strength (increase 1.41 times) and an improved elongation at break (increase 2.53 times) compared to the control film. Although the antioxidant activity of GO-containing films showed a slight decline, the antibacterial performance was considerably enhanced, particularly against *E. coli*, with the inhibition zone diameter increasing by 26.66–35.84% compared to the control film. Experimental storage of fresh plums showed that the CH–GO film helped maintain fruit freshness and gloss, preventing spoilage for up to 10 days. Additionally, after 10 days, the film reduced weight loss (by 21.60–72.53% compared to the pure chitosan film group and 16.10–42.63% compared to the control group), slowed color changes, stabilized pH, maintained soluble solid content (reducing the loss by up to 10.54% compared to the control group), and preserved total acidity throughout the storage period. Based on these findings, CH–GO composite films can be considered a promising packaging material for the preservation of fresh produce.

## Abstract 2

CH–GO composite films enhance mechanical strength, barrier, and antibacterial properties, effectively extending the shelf life and freshness of plums.

## Graphical abstract

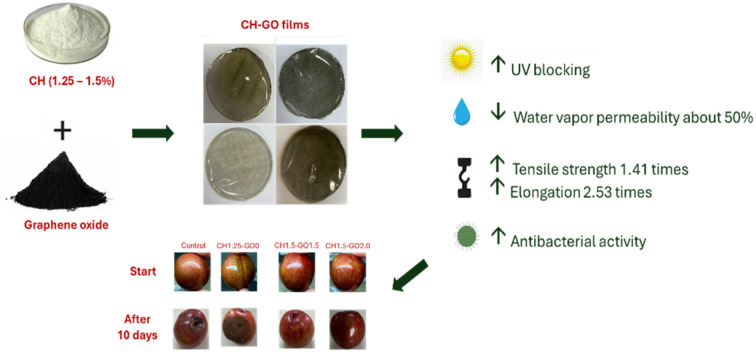

## 1 Introduction

Fresh fruits contain a large number of compounds beneficial to human health, including antioxidants, anthocyanins, phenolic compounds, and various vitamins, which help reduce the risk of chronic diseases and promote overall well-being.^1^ Therefore, fresh fruits are considered one of the essential food groups in the daily diet. In particular, the plum (*Prunus salicina* Lindl.), belonging to the Rosaceae family, is a fruit with a distinctive flavor and contains relatively higher levels of valuable bioactive compounds such as anthocyanins, polyphenols, ascorbic acid, and β-carotene compared to many other fruits.^2–4^ Consequently, plums exhibit potent antioxidant and anti-inflammatory properties. In addition, due to their rich nutritional composition, plums offer several health benefits, including acting as a natural laxative, improving gastric function, enhancing memory and cognitive performance, promoting bone health, and reducing the risk of cardiovascular diseases.^5^ Moreover, the presence of components such as malic acid, sugars, pectin, amygdalin, and prunasin in plums contributes to supporting digestion, reducing muscle fatigue, providing energy, stabilizing blood sugar levels, and improving mood.^4^ However, this fruit is mainly cultivated in certain Asian countries such as China and Vietnam, as well as in some European regions. However, this fruit is mainly cultivated in certain Asian countries such as China and Vietnam, as well as in some European nations. It is a seasonal fruit with significant health benefits and high economic value for growing regions. Statistics indicate that the annual plum production reaches approximately 6.7 million tons in China, over 6.7 thousand tons in Romania, and nearly 500 thousand tons in Serbia.^6^ With such a large production volume, it is challenging to consume all the plums locally, leading to a decrease in their commercial value. Moreover, plums have a short harvesting period, thin skin, and high-water content, making them highly susceptible to spoilage caused by fungal contamination or mechanical damage during transportation and storage. Postharvest losses of plums are estimated to reach up to 800 million USD annually, accounting for approximately 35–40% of the total yield.^4^ Without proper preservation and distribution methods, a significant portion of the harvested plums would be wasted. In addition, transportation to distant regions requires considerable time; therefore, effective preservation techniques are essential to maintain the quality, nutritional value, and bioactive compounds of plums over an extended period, meeting market demand and minimizing losses for farmers.

Currently, various preservation methods are applied to fresh fruits in general and plums in particular, such as the use of chemical treatments, cold storage, irradiation, or packaging materials. However, chemical preservation poses the risk of toxic residue that may affect human health; cold storage can damage fruit tissue structures, resulting in soft texture and flavor loss; while irradiation methods are costly.^7^ Therefore, the use of naturally preservative packaging materials is considered the safest and most economical approach at present.

However, most packaging materials currently in use are plastics derived from petroleum – a resource that is gradually being depleted. Furthermore, the global demand for plastic packaging in the food industry continues to rise rapidly due to its mechanical advantages and low production cost.^8^ To date, plastic packaging has caused severe environmental problems such as climate change, the greenhouse effect, and pollution due to its poor biodegradability and limited recyclability.^9,10^ To mitigate these negative impacts, the development of biodegradable packaging materials is considered a promising solution.^11^ In addition, current consumer trends increasingly favor packaging that is safe, health-friendly, and naturally sourced. Driven by these practical demands, the research and development of biodegradable bio-based packaging films with high safety and environmental compatibility has become an area of great interest among scientists worldwide.

With the growing trend toward developing environmentally friendly and health-safe packaging materials, chitosan has attracted considerable attention in the development of bio-based films due to its excellent film-forming ability.^9,12^ Chitosan is a natural polymer primarily derived from the shells of crustaceans or fungal cell walls and is the second most abundant natural polymer after cellulose.^13,14^ In addition to its abundance and availability, chitosan possesses several remarkable properties such as biocompatibility, biodegradability, and safety for both human health and the environment.^9,15^ Thanks to these advantages, chitosan has been widely applied in various fields, including medicine (for wound healing, tissue regeneration, and bone replacement), wastewater treatment (particularly for oil and heavy metal removal), biosensors,^15^ and especially as biofilms for food preservation.^16,17^ However, chitosan-based films have several limitations, such as poor mechanical strength (easily broken, low tensile strength), high water absorption, and high solubility in humid environments,^9^ which restrict their use in food packaging applications. To overcome these drawbacks, many studies have proposed enhancing the properties of chitosan by incorporating reinforcing agents such as GO, carbon nanomaterials, or metal oxides (*e.g.*, ZnO, TiO_2_). These additives not only improve mechanical strength and thermal stability but also enhance the antibacterial properties of the films.^18,19^

Among these, graphene oxide (GO), a nanomaterial composed of two-dimensional carbon sheets – has garnered particular interest in recent years.^15^ GO is typically synthesized through the oxidation of graphite;^9,20^ and is known for its outstanding mechanical strength, electrical conductivity, biodegradability, and biocompatibility.^21^ Notably, GO has been shown to exhibit potent antibacterial activity,^22^ making it a promising material for biomedical and food packaging applications. The development of antimicrobial packaging is a promising approach for actively controlling microbial growth, thereby extending shelf life, improving food quality, and ensuring food safety.^8,23^

Recent studies have demonstrated that combining chitosan and GO produces composite materials with superior mechanical and thermal properties and enhanced antibacterial activity. These composites have shown potential applications in construction materials (*e.g.*, cement),^24^ materials,^25^ biomedical engineering,^9,26^ and wastewater treatment among others.^27^ In the field of food packaging and preservation, chitosan–graphene oxide (CH–GO) films have been tested for the storage of margarine, demonstrating their suitability for preserving fat-containing products.^28^ In addition, a composite film based on polyvinyl alcohol/CH/GO/silver has been successfully developed and applied in the preservation of green plums, effectively blocking UV radiation for up to 10 days. The results revealed that this composite film exhibited significantly improved tensile strength, reduced water swelling, enhanced thermal stability, and higher antibacterial activity compared with pure PVA films.^29^ However, the incorporation of silver nanoparticles (AgNPs) considerably increases production costs due to the high price of the material, and the synthesis process requires NaBH_4_ as a reducing agent, further adding to production expenses. Moreover, the potential residual heavy metals (*e.g.*, silver, titanium) in food packaging raise safety concerns among consumers. Biodegradable CH–GO bags have also been investigated for the preservation of mangoes and melons, showing that CH–GO films can effectively delay ripening, maintain external appearance, and exhibit promising preservation effects for thick-skinned fruits.^30,31^ Nonetheless, previous studies have generally been limited to a single chitosan concentration and a fixed GO content, without comprehensively assessing how different CH : GO ratios affect the film's physicochemical properties and preservation performance. Furthermore, most existing research has focused on large, thick-skinned fruits with low water loss and long storage periods. In contrast, there are very few studies evaluating the effectiveness of CH–GO films in preserving more “sensitive” fruits, such as red plums, which have a short shelf life, small size, thin peel, and high moisture content. Additionally, current publications on CH–GO materials mainly focus on isolated properties, while systematic investigations simultaneously examining the effects of varying GO concentrations on key characteristics – such as mechanical, optical, and antibacterial properties as well as actual preservation efficiency – remain limited.

Therefore, in this study, the mechanical properties together with preservation-related characteristics such as antioxidant and antibacterial activities of chitosan films at different concentrations, incorporated with various amounts of GO, were comprehensively investigated and evaluated. The film characteristics were analyzed using scanning electron microscopy (SEM), Fourier-transform infrared spectroscopy (FTIR), surface roughness measurements, optical properties, and colorimetric analysis. In addition, the biological activities, including antibacterial and antioxidant capacities, were also determined. Furthermore, the mechanical properties (such as tensile strength and elongation at break), water absorption, and solubility of the films were evaluated. Notably, for the first time, CH–GO films with different compositional ratios were applied to the preservation of fresh plums. The findings of this study aim to develop packaging materials with strong antibacterial, antioxidant, and mechanical properties, as well as excellent UV-barrier capacity, thereby enhancing the preservation efficiency of fresh fruits such as plums. This research not only contributes to identifying novel bio-based packaging materials as alternatives to conventional plastics but also promotes sustainable postharvest preservation practices, helping to reduce losses and waste of agricultural products.

## 2 Materials and methods

### 2.1. Materials and methods, crystallography

Chitosan was purchased from Chitosan Vietnam Company (Ho Chi Minh City, Vietnam) in the form of fine powder, off-white in color, moisture content <10%, ash: 0.56%, purity ≥ 90%, and DE ≥ 90.0%. Graphite powder was obtained from Tianjin Dengke Chemical Reagent Company (China), the purity: 98%. Several chemicals used in this study included: acetic acid – the purity: 99.5% pure (Xilong, China), glycerol: the concentration: 99% (Xilong, China), DPPH (2,2-diphenyl-1-picrylhydrazyl) (Sigma, Germany), agar (Himedia, India), peptone (Merck, Germany), NaCl – the purity: 99.5% (Vietchem, Vietnam), KMnO_4_ – the purity: 99.0% (Merck, Germany), hydrogen peroxide 30% (Xilong, China), HCl 30–35% (Xilong, China), and H_2_SO_4_ 95–97% (Xilong, China).

### 2.2. Synthesis of graphene oxide (GO)

GO was synthesized based on a previously published method with several modifications to suit the practical conditions of this study.^28^ Graphite was added to concentrated H_2_SO_4_ at a ratio of 1 : 80 g mL^−1^, placed in an ice bath, and stirred continuously for 30 min. Then, 6 g of KMnO_4_ was gradually added to the mixture while maintaining continuous stirring. The mixture was subsequently stirred at a temperature of 30–35 °C for 24 h. After that, 100 mL of distilled water was added, and stirring continued for another 30 min. Next, 100 mL of H_2_O_2_ solution (1 : 25 v/v) was slowly added to the mixture and stirred for 10 min. The resulting mixture was filtered to collect the precipitate, which was then washed with HCl solution, followed by distilled water until a neutral pH was achieved. The final precipitation was redispersed in water and subjected to ultrasonication in a bath sonicator for 4 h. The resulting suspension was then dried to obtain GO in fine powder form.

### 2.3. Preparation of CH–GO films

The method for synthesizing CH films incorporated with GO was prepared based on the description by Wrońska and *et al.* with modification:^9^ Chitosan solutions were prepared by dissolving chitosan in 1% (v/v) acetic acid solution at concentrations of 1.25% (w/v) and 1.5% (w/v), with continuous stirring at 50 °C for 2 h. GO at concentrations of 0, 0.5, 1.0, 1.5, and 2.0% (w_GO_/w_CH_) was dispersed in 2 mL of 1% acetic acid solution and sonicated at 50 °C for 1.5 h.^27^ The two prepared mixtures were then mixed, stirred thoroughly with 20% glycerol (w/w), and continuously stirred for 1.5 h. The final mixture was molded and dried at temperature 50 °C for 2 days until a constant weight was achieved.

### 2.4. Characteristics of CH–GO films

#### 2.4.1. FTIR

The functional groups present in the films were analyzed using a Fourier-transform infrared (FTIR) spectrometer, Nicolet iS10 (Thermo Scientific, USA), the wavenumber range of 4000–400 cm^−1^, and the speed of 40 scans per s.

#### 2.4.2. SEM

The surface morphology of the films was examined using a scanning electron microscope (SEM) model JMS-6510LV (JEOL, Japan) with a magnification range of 100 times and an accelerating voltage of 20 kV.

The surface roughness of the films was analyzed using Gwyddion software version 2.68.

#### 2.4.3. Thickness

Film thickness was measured using a digital caliper (Mitutoyo 500-181-30, Kawasaki, Japan) at five different positions on each film.

#### 2.4.4. Colors

The color of the materials was measured using a handheld colorimeter Lovibond LC-100 (Lovibond, China). The reference standard was white, with characteristic values of L*: 98.5, a*: −0.4, and b*: 0.5. The color saturation (C*), whiteness index (WI), and yellowness index (YI) were calculated using eqn (1)–(3):^32,33^1
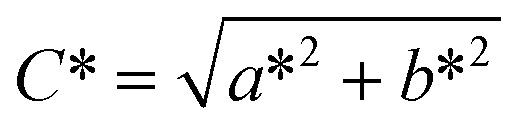
2
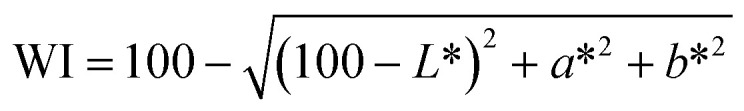
3
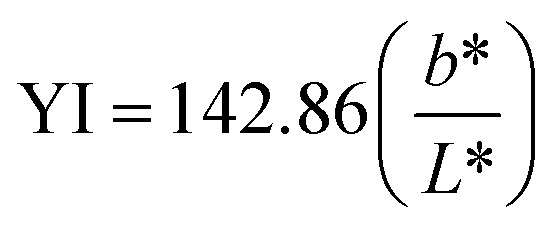


#### 2.4.5. Light transmittance and opacity

The light transmittance of the materials was measured using a Nabi UV/VIS Nano Spectrophotometer (Nabi, South Korea) in the wavelength range of 360 to 700 nm. Films were prepared with dimensions of 4.8 × 4 cm and evenly placed in cuvettes. The transmittance (*T*, %) of the samples was recorded, and the opacity was calculated using eqn (4):^28^4
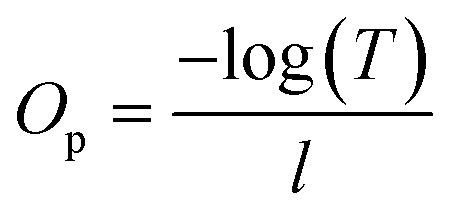
where, *O*_p_: the opacity of film; −log(*T*): absorbance at 600 nm wavelength; *l*: the film thickness, mm.

#### 2.4.6. Water vapor permeability

Water vapor permeability was determined according to the ASTM E96 method.^34^ Specifically, a glass vial containing 10 mL of water was sealed at the mouth with a pre-measured film sample. The vial was then placed inside a desiccator maintained at a relative humidity of 75 ± 3%, using silica gel as the desiccant. The weight of the vial was recorded every hour for a total of 6 h. The water vapor permeability was calculated using eqn (5):^3,28^5
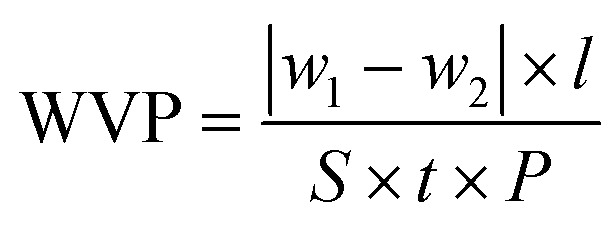
where, *w*_1_, *w*_2_: weight of the glass vial before and after the test, g; *l*: film thickness, m; *S*: surface area of the film, m^2^; *t*: time, h; Δ*P*: partial pressure difference of water vapor across the film, Pa.

#### 2.4.7. Solubility

The solubility of the film was determined by preparing a film sample of known weight and size (2 × 2 cm), which was dried at 60 °C until a constant weight was achieved. The dried films were then immersed in water at room temperature for 24 hours. After immersing, the films were removed and dried at 60 °C until a constant weighed. The solubility of the film was calculated by eqn (6):^3^6
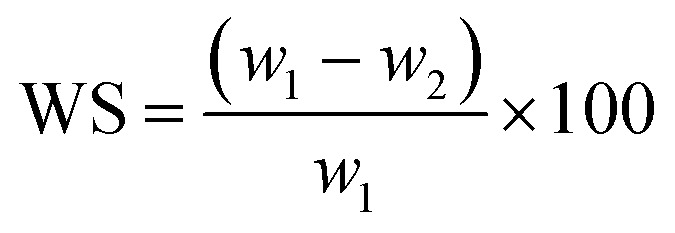
where, WS: solubility of film after 24 h, %; *w*_1_: the initial weight of film, g; *w*_2_: weight of film after immersing 24 h, g.

#### 2.4.8. Water absorption capacity

To determine the water absorption capacity, films with dimensions of 2 × 2 cm were prepared and weighed. Then, the films were immersed in water for 5, 10, 15, 20, 30, 60, 90, and 120 min. After each soaking period, the films were removed, drained to remove excess water, and weighed. The water absorption capacity was calculated using eqn (7):7
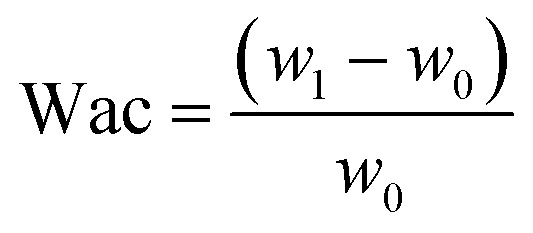
where, Wac: the water absorption capacity, g g^−1^; *w*_1_: weight of film after soaking, g; *w*_o_: the initial weight of film, g.

#### 2.4.9. Antioxidant activity

The antioxidant activity of the films was determined using the DPPH method.^28^ About 0.01 g film was dissolved in 5 mL of ethanol in 1 h. Then, 1.5 mL of this solution was mixed with 1.5 mL of 0.6 mM DPPH solution, shaken thoroughly, and incubated in the dark for 60 min at room temperature. The absorbance was measured at a wavelength of 517 nm. The blank sample consisted of ethanol mixed with 0.6 mM DPPH solution. The DPPH radical scavenging activity was calculated using eqn (8):8
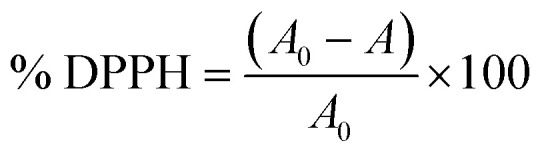
where, % DPPH: the DPPH radical scavenging activity of film, %; *A*_0_: absorbance of blank sample; *A*: absorbance of film.

#### 2.4.10. Antibacterial activity

The antibacterial activity of the prepared films was evaluated using the agar diffusion method. Two representative bacterial strains, *Escherichia coli* (Gram-negative) and *Staphylococcus aureus* (Gram-positive), were employed in this study. Luria–Bertani (LB) agar medium was poured into Petri dishes and allowed to solidify. Subsequently, 100 µL of bacterial suspension with a concentration of 10^6^ CFU mL^−1^ was evenly spread on the agar surface. Film samples with a diameter of 5 cm were prepared and placed directly onto the inoculated agar surface. The plates were then incubated under appropriate conditions. After incubation, the inhibition zones surrounding the films were observed and measured. The diameter of the inhibition zone was used to assess the antibacterial effectiveness of the films.^35^

#### 2.4.11. Mechanical properties

Film samples were prepared with a size of 1 × 10 cm. Tensile strength and elongation at break were then analyzed according to ASTM D882 using a universal testing machine AGX-50kNVD (SHIMADZU, Japan) with a capacity of 50 kN and a crosshead speed of 10 mm min^−1^.^36^

### 2.5. Plum preservation using the CH–GO films

The plums were sourced from local farming households. The selected fruits were physiologically mature, with signs including skin color beginning to change from green to light red or showing reddish-purple spots. The fruits remained firm and were not overly soft. After harvesting, the plums were transported to the laboratory, where fruits with uniform size and color were selected for preservation experiments. The fruits were washed with clean water, drained, and divided into different groups for storage. A total of 9 experimental groups were prepared, including:

Group 1 (control group): fruits were not treated with any preservation method.

Groups 2 to 9: fruits were preserved using different types of CH–GO films. The film-forming solutions were prepared and cooled before use. The fruits were immersed in the solution for 2 min, air-dried for 15 min, and then immersed again for another 2 min before final drying. All the formulated films were used in the preservation study.

Each group contained 5 fruits. All fruit groups were stored at 25 ± 2 °C the relative humidity (RH) ranged from 55% to 65% for 10 days. During the storage period, every 2 days, the following parameters were analyzed and recorded: fruit condition description, weight loss percentage, total soluble solids (TSS), pH, titratable acidity, and fruit color.

#### 2.5.1. Evaluation of external appearance

The visual quality of plum appearance was assessed using a 9-point hedonic scale, based on color, surface condition, firmness, and the presence of decay or dehydration.^30^ The scoring criteria were as follows Table 1.

**Table 1:** Nine-point scale for evaluating the external appearance of plums

Point	Description
9 (Excellent)	Plums are extremely fresh with perfect appearance; smooth, glossy, and firm surface when gently pressed; no spots, wounds, or signs of decay
8 (Very good)	Fresh plums with very attractive appearance; slightly reduced surface gloss and tightness; still firm to touch; no visible spots or decay
7 (Good)	Fresh plums with good appearance; slightly reduced gloss and tightness (about 5% compared to the initial state); firm when pressed lightly; no visible spots or defects
6 (Fair)	Normal appearance; a few small spots (about 5%) or slight wrinkles (about 5% of the surface area) appear; firmness decreases when pressed
5 (Acceptable)	Noticeable decline in external appearance; about 10% of the surface shows spots or wrinkles; surface gloss starts to fade; still edible
4 (Poor)	Unattractive appearance; about 25% of the surface shows spots, wrinkles, or bruises; fruit begins to soften when pressed
3 (Very poor)	Very unattractive appearance; about 50% of the surface shows spots, wrinkles, and/or slight decay; noticeably soft to the touch
2 (Extremely poor)	Extremely unattractive; about 75% of the surface shows spots, wrinkles, or soft and decayed areas; signs of mild rot
1 (Completely spoiled)	Severely decayed; completely soft and discolored; over 75% of the surface shows dark rot or spoilage; unfit for consumption

The evaluation was carried out by five trained panelists, each assessing independently, and the average score was used for statistical analysis.

#### 2.5.2. Weight loss percentage

The weight loss percentage was calculated using eqn (9):^17,21^9
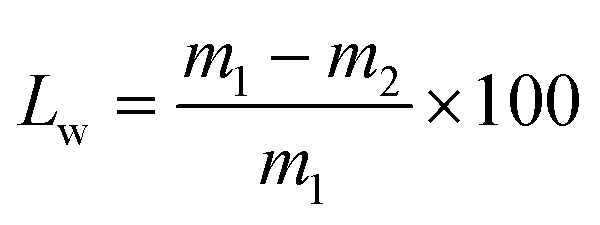
where, *L*_w_: weight loss percentage, %; *m*_1_: the initial weight of fruit, g; *m*_2_: the weight of fruit after preservation, g.

#### 2.5.3. Color of fruits

The color of the fruit was measured using a Lovibond LC-100 colorimeter (Lovibond, China), with white as the reference standard. The Δ*E* value was recorded and used to calculate the color difference compared to the initial color of the fruit.

Juice from the fruits was collected to determine TSS, titratable acidity, and pH.

#### 2.5.4. Total soluble solids (TSS), and pH

TSS was measured using a refractometer (Master-T, Atago, Japan) and presented in Brix (°Bx).^16,21^

pH was measured using a benchtop pH meter ST2200-F Starter 2200 (Ohaus, USA).

#### 2.5.5. Titratable acidity

Titratable acidity was determined using acid–base titration: a 5 mL juice sample was mixed with 15 mL distilled water and titrated with 0.1 N NaOH using 1% phenolphthalein as an indicator until a persistent pale pink color appeared for 15 seconds. The acidity content was calculated using eqn (10):^1,37^10
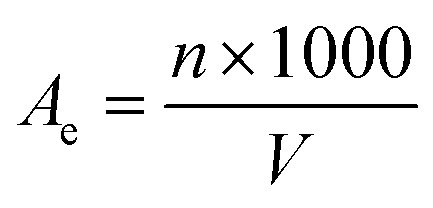
where, *A*_e_: The acidity content, mqE L^−1^; *V*: the volume of juice used for the experiment, mL; *n*: the volume of 0.1 N NaOH used for titration, mL.

### 2.6. Statistical analysis

All data were collected in three replicates or experimental units per treatment, and results are presented as mean ± standard deviation (SD). The experiments were arranged in a completely randomized design (CRD). Two-way analysis of variance (ANOVA) was performed using Minitab software version 16.0.0.2 to compare differences among means. Tukey's test was used for analysis to determine statistically significant differences at a significance level of *p* < 0.05. In addition, Gwyddion software was used for 3D surface image simulation of the studied films.

## 3 Results and discussion

### 3.1. Film-forming ability

Two CH concentrations, 1.25% and 1.5%, were selected based on preliminary tests conducted within the range of 1.0–2.0%. At concentrations below 1.0%, the resulting films were very thin, the film-forming process was complicated, and it was challenging to obtain intact films. Conversely, at a concentration of 1.5%, the film-forming solution became too viscous and dense, leading to films with non-uniform thickness. Therefore, the two concentrations of 1.25% and 1.5% CH were chosen for further evaluation in this study.

**Fig. 1:**
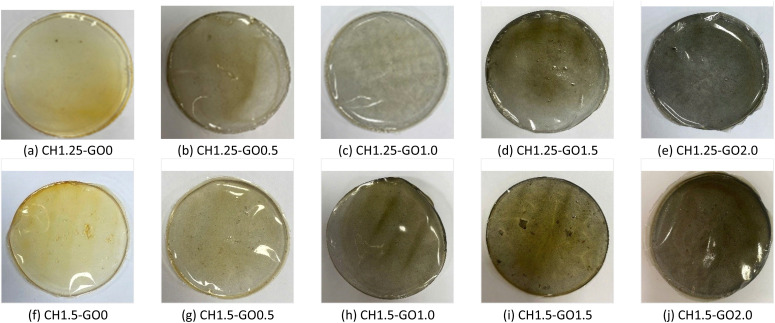
Actual appearance of the CH–GO films. (a) CH1.25–GO0, (b) CH1.25–GO0.5, (c) CH1.25–GO1.0, (d) CH1.25–GO1.5, (e) CH1.25–GO2.0, (f) CH1.5–GO0, (g) CH1.5–GO0.5, (h) CH1.5–GO1.0, (i) CH1.5–GO1.5, (j) CH1.5–GO2.0.

The observed results of the actual film formation using different CH concentrations (1.25% and 1.5%) and various ratios of GO (from 0% to 2%) were presented in Fig. 1. The results showed that for both CH concentrations, as the amount of GO added to the film increased, the film color changed from the original yellow of chitosan to gray, dark gray, or brownish-black, especially when the GO content was 1.5%. In addition, higher GO content led to increased opacity and decreased transparency of the films. When the GO content exceeded 1.5%, aggregation of GO particles became visible on the film surface due to uneven dispersion.

Regarding CH concentration, increasing the CH content resulted in thicker, darker, and more opaque films compared to those with lower CH (1.25%). This was likely since a higher CH increased the viscosity of the film-forming solution, which in turn resulted in thicker films at higher concentrations.

Based on visual observation, the application of these films in food packaging requires appropriate selection depending on the product type. Films such as CH1.25–GO0.5, CH1.25–GO1.0, and CH1.5–GO0.5, which were more transparent and less opaque, were suitable for packaging fresh foods where visual appearance was important. In contrast, darker-colored films were more appropriate for packaging or preserving dark-colored fruits or food products that were sensitive to light exposure, such as cheese, dried sausages, dark-colored pastries, or powdered nutritional products.

### 3.2. FTIR

**Fig. 2:**
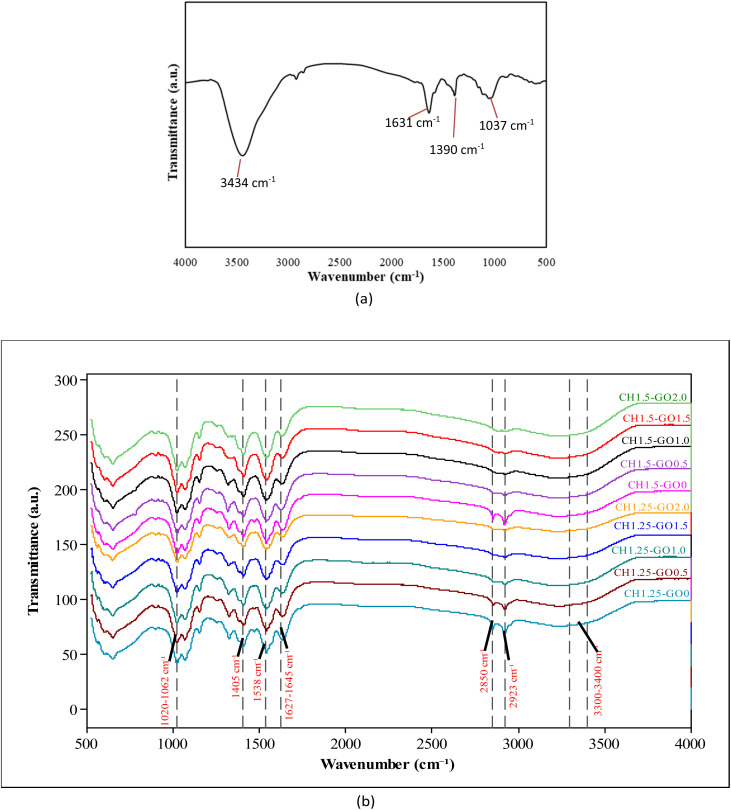
FTIR spectra of (a) GO and (b) CH–GO composite films.

The FTIR spectrum of GO shown in Fig. 2a exhibits a broad absorption band at 3434 cm^−1^, corresponding to the stretching vibration of hydroxyl (–OH) groups.^38,39^ The characteristic peaks at 1631 cm^−1^, 1390 cm^−1^, and 1037 cm^−1^ are assigned to the C=C stretching vibration of the unoxidized GO framework, the C–OH stretching vibration, and the C–O–C stretching vibration of epoxy groups, respectively. The bands observed at 2927 cm^−1^ and 2849 cm^−1^ correspond to the asymmetric and symmetric stretching vibrations of methylene (CH_2_) groups present in the GO structure.^40^ The FTIR peaks identified in this study are consistent with previously reported spectra of GO,^38,41^ confirming that the oxidation of graphite introduced various oxygen-containing functional groups such as hydroxyl, epoxy, and carboxyl. However, in this study, the –OH peak intensity is noticeably higher than in previous reports, indicating a greater abundance of hydroxyl groups on the GO surface. This may be attributed to differences in oxidation conditions or to a longer reaction time, which could have promoted the attachment of additional hydroxyl groups to the carbon framework.^40^ Conversely, the peaks corresponding to CH_2_ stretching vibrations appear with lower intensity, suggesting that the oxidation process may have disrupted aliphatic hydrocarbon chains on the GO surface, breaking them into smaller fragments. The high content of oxygen-containing functional groups, particularly –OH and C–O–C, is expected to enhance the interfacial interaction between GO and the CH polymer matrix.

The FTIR spectra of both pure CH and the CH–GO composite films are presented in Fig. 2. The broad band in the range of 3200–3500 cm^−1^ corresponds to hydroxyl stretching vibrations from –OH and amine groups in chitosan.^39^ The intensity of this band gradually decreases with increasing GO content, indicating that GO participates in hydrogen bonding interactions with the hydroxyl and amine groups of CH. The decrease in intensity, accompanied by a slight shift toward higher wavenumbers, suggests the formation of weaker hydrogen bonds, possibly due to the partial saturation of chitosan's –OH groups through interactions with the GO surface. This contributes to the formation of a more stable structural network between the two material phases.

Two peaks at 2850 cm^−1^ and 2923 cm^−1^, corresponding to C–H stretching vibrations, are clearly visible in the pure CH film and in the sample containing 0.5% GO. Still, they diminish as the GO concentration increases. This reduction indicates a decrease in the density of free C–H groups due to interfacial interactions between CH chains and GO, which restricts the free vibration of the polymer backbone. The peaks in the region of 1645–1627 cm^−1^ correspond to amide I or carbonyl groups from acetylated amino groups present in CH.^38,39^ The intensity of this band decreases progressively with increasing GO content, attributed to hydrogen bond formation between GO and the hexagonal rings of CH.^38,39^

The peaks at 1538 cm^−1^ and 1405 cm^−1^, corresponding to N–H bending (amide II) and C–OH stretching vibrations, respectively, are also observed in the spectra of the films.^39^ However, the intensities of these peaks remain nearly unchanged with varying CH and GO ratios. The peaks observed at 1020–1062 cm^−1^ are attributed to the C–O–C stretching vibrations from GO layers.^38,39^ The absence of new peaks in the FTIR spectra of CH–GO films indicates that GO interacts mainly through selective physical interactions – such as hydrogen bonding and π–π stacking – with the functional groups of CH, rather than forming new chemical bonds or altering the polymer's chemical structure. These interactions enhance adhesion and dispersion between the two components, leading to improved mechanical properties and structural stability of the CH films containing GO compared to pure CH films.

### 3.3. Surface morphology

**Fig. 3:**
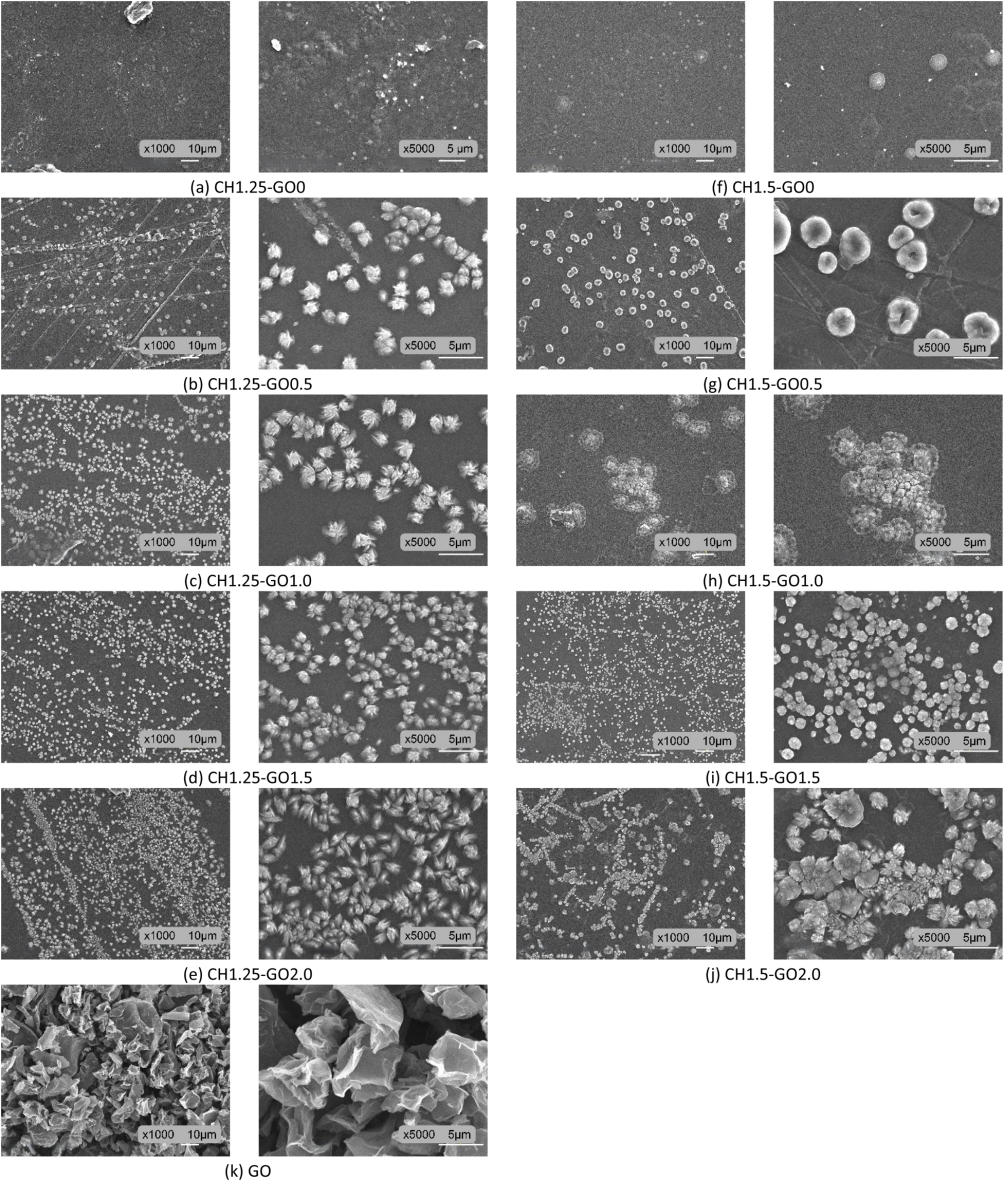
SEM of CH–GO films. (a) CH1.25–GO0. (b) CH1.25–GO0.5. (c) CH1.25–GO1.0. (d) CH1.25–GO1.5. (e) CH1.25–GO2.0. (f) CH1.5–GO0. (g) CH1.5–GO0.5. (h) CH1.5–GO1.0. (i) CH1.5–GO1.5. (j) CH1.5–GO2.0. (k) GO.

The surface morphology of GO and the bio-based films was analyzed using scanning electron microscopy (SEM) and presented in Fig. 3. The results showed that the pure CH film exhibits a smooth and homogeneous surface, which is characteristic of a polymer structure without phase interaction. Upon the incorporation of GO, the appearance of distinct GO aggregates visibly altered the surface morphology, resulting in increased roughness and heterogeneity. This change reflects the physical interactions between GO sheets and the CH polymer matrix, which may generate localized stress regions and enhance mechanical bonding between the two material phases.

At a GO concentration of 0.5%, the GO layers are relatively well dispersed within the CH matrix, indicating favorable interactions between the functional groups of chitosan (–NH_2_, –OH) and the oxygen-containing groups on the GO surface. This uniform distribution not only improves the mechanical properties and thermal stability of the film but also suggests the formation of a well-balanced hydrogen-bonding network between GO and the CH polymer.^42^

However, at concentrations above 1.0%, evident GO agglomeration occurs, leading to denser accumulation of GO particles on the film surface. Particularly at 2.0% GO, the film structure is dominated by compact GO clusters, while the CH matrix becomes significantly reduced. This phenomenon may result from strong van der Waals interactions between GO sheets, which overpower their interactions with the polymer matrix. These findings suggest that exceeding the optimal concentration (0.5%) leads to poor GO dispersion, which may hinder stress transfer and consequently weaken the mechanical performance of the resulting films. A similar trend was also reported by Ahmed.^42^

The SEM image of the GO shows that it consists of multiple layers formed by flat, sheet-like fragments. These fragments are relatively large, which can affect the dispersion of GO within the polymer matrix. This structure is similar to that previously reported for GO;^27,43^ however, the GO surface in this study appears flatter and exhibits more distinct layering than that reported by Zhanna.^43^

**Fig. 4:**
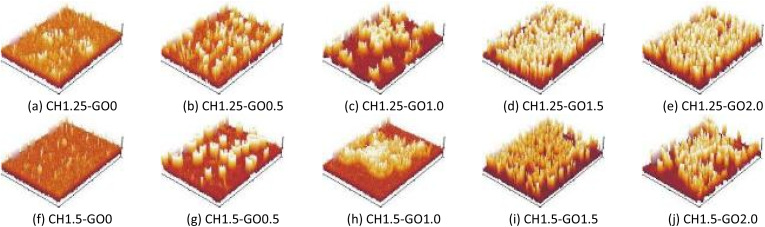
The surface roughness of CH–GO films. (a) CH1.25–GO0. (b) CH1.25–GO0.5. (c) CH1.25–GO1.0. (d) CH1.25–GO1.5. (e) CH1.25–GO2.0. (f) CH1.5–GO0. (g) CH1.5–GO0.5. (h) CH1.5–GO1.0. (i) CH1.5–GO1.5. (j) CH1.5–GO2.0.

Fig. 4 shows the 3D surface roughness simulations of the studied films. The results demonstrate a significant difference in surface roughness between pure CH films and CH films containing GO. Specifically, the films without GO exhibited flat, smooth surfaces with few, low, and sparsely distributed peaks. This could be attributed to slight agglomeration or uneven dispersion of CH in the film-forming solution. Nevertheless, the overall uniform surface of the pure CH film suggests a well-organized polymer network. As GO was introduced, the number of peaks increased, indicating enhanced surface roughness. The 3D data showed that at low GO content (0.5%), the GO particles were sparsely distributed and surrounded by the polymer matrix, forming a stable structure. However, when the GO content exceeded 1.0%, the peaks became more clustered, taller, and more densely packed (Fig. 4c–e and h–j). Particularly, with GO content in the range of 1.5–2.0%, the film surface was dominated by large GO clusters with significantly increased peak heights, which reduced the surface uniformity of the films.

In summary, the incorporation of GO into the CH films significantly increased surface roughness, thereby enlarging the surface area of the films. This observation is consistent with previous findings reported by Ahmed^42^ and Yang.^44^

### 3.4. Film thickness

**Fig. 5:**
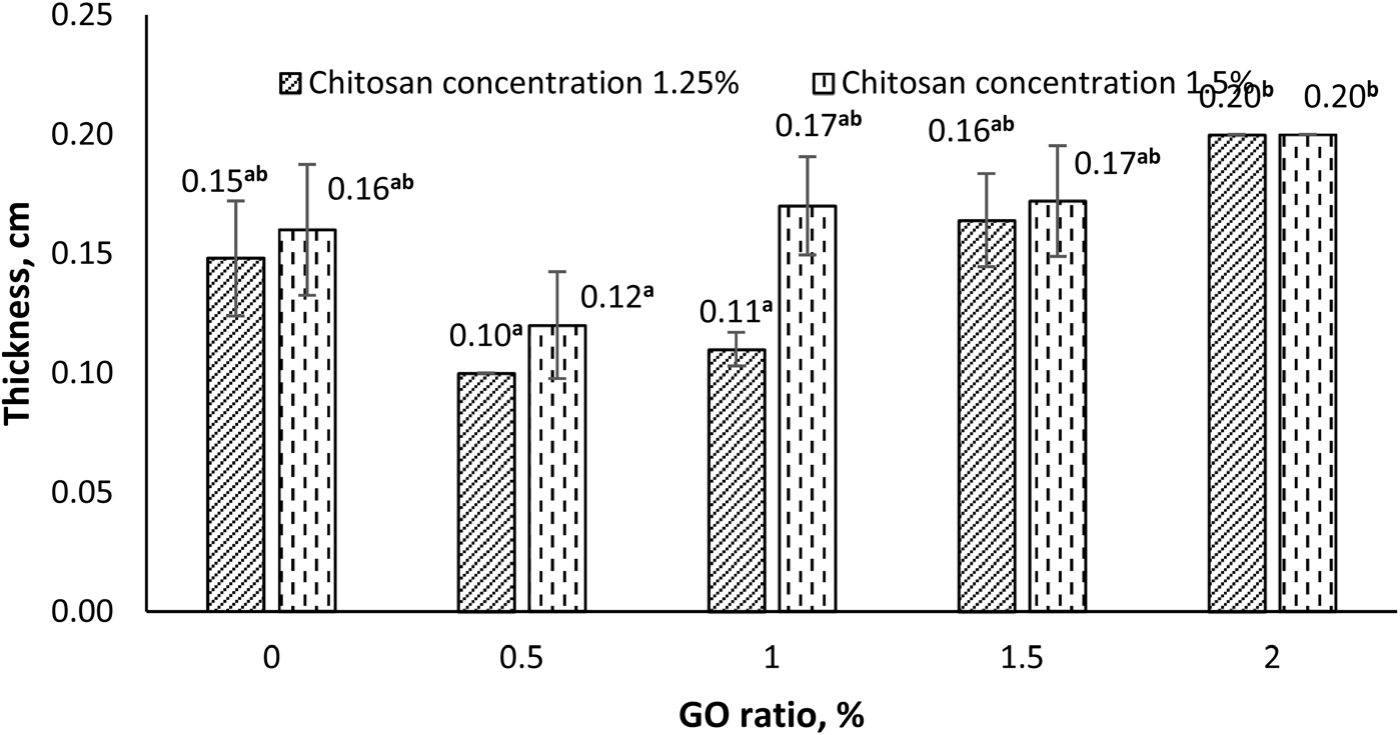
Effect of CH ratio and GO content on film thickness (Data were analyzed using two-way ANOVA at a significance level of 0.05, where *P*-value (CH) = 0.056 > 0.05, *P*-value (GO) = 0.000 < 0.05, and *P*-value (CH × GO) = 0.385 > 0.05, based on Tukey's test).

The thickness of the films prepared from CH with varying GO contents was presented in Fig. 5. The results indicated that films with higher CH concentrations generally exhibited greater thickness. However, when GO was added at ratios ranging from 0.5% to 1.5% into films containing 1.25% chitosan, the resulting film thickness was lower compared to those made with 1.5% chitosan. When the GO content increased to 2.0%, changes in CH concentration no longer had a significant effect on film thickness. In addition, an increase in GO content tended to increase the film thickness. Nevertheless, at low GO ratios (about 0.5%), the resulting films were thinner than the blank CH film. This result was also clearly observed in the visual images of the films shown in Fig. 1.

The statistical analysis results showed that the concentration of CH and the interaction between CH and GO had no statistically significant effect (*p* > 0.05) on the thickness of the obtained films. In contrast, the amount of GO added had a considerable influence (*p* < 0.05). In other words, the film thickness was mainly determined by the GO content incorporated into the polymer matrix. In contrast, the effect of GO concentration on film thickness was not significantly dependent on the CH level used in the film-forming formulation.

The variation in the thickness of CH films with different GO contents can be explained by the molecular rearrangement between GO and CH at low GO concentrations, which leads to thinner films because GO is lightweight and has a porous structure. However, when the GO concentration becomes too high, the GO network becomes denser, reducing the intermolecular spacing and resulting in a more compact molecular arrangement and thicker films. The oxygen-containing functional groups of GO (–COOH, –OH, –C=O) can form hydrogen bonds with the amino and hydroxyl groups of CH, enhancing polymer–polymer interactions and leading to stronger and thicker film structures. Nevertheless, at high GO concentrations, the aggregation of GO sheets may occur, causing uneven dispersion and reducing film uniformity.

However, a previous report on CH films incorporated with GO revealed that the CH ratio and GO content did not significantly affect the obtained film thickness.^28^ This discrepancy may be attributed to differences in casting techniques. Overall, the thickness of the CH–GO films in this study ranged from 1–2 mm, which is higher than those reported by Han *et al.* (79.70–87.40 µm),^28^ Ahmed (0.172–0.177 mm),^42^ and Vilvert *et al.* (49.0 µm).^30^ Such differences may be related to the volume of film-forming solution used and the different material synthesis techniques employed.

From these results, it can be concluded that adjusting the GO content within an appropriate range can help control and optimize the thickness of CH–GO films during material fabrication to suit different applications and practical conditions.

### 3.5. Color

The color changes of CH films incorporated with different concentrations of GO are presented in Table 2. The results show that the lightness (L*) of the films decreases with increasing GO content. Specifically, when the GO content increases from 0.5% to 1.0%, the decrease in lightness is slight; however, at contents above 1.0%, the reduction becomes more pronounced. This can be attributed to the inherently dark color of GO, which darkens the overall appearance of the films.

**Table 2:** The color results of CH–GO films

Sample	L*	a*	b*	Δ*E*	C*	WI	YI
CH1.25–GO0	55.87 ± 0.87^a^	9.13 ± 0.75^a^	25.43 ± 0.84^a^	50.31 ± 0.68^e^	27.03 ± 0.59^a^	48.24 ± 0.69^a^	65.04 ± 2.19^a^
CH1.25–GO0.5	42.50 ± 1.23^cd^	3.27 ± 0.46^cde^	8.43 ± 0.86^e^	56.68 ± 1.30 ^cd^	9.06 ± 0.64^de^	41.79 ± 1.31^bc^	28.42 ± 3.67^de^
CH1.25–GO1.0	41.53 ± 1.15^d^	3.63 ± 0.38^c^	8.33 ± 0.64^e^	57.65 ± 1.19^c^	9.10 ± 0.50^de^	40.83 ± 1.19^c^	28.70 ± 2.68^d^
CH1.25–GO1.5	35.50 ± 0.96^e^	3.70 ± 0.20^c^	8.07 ± 0.67^e^	63.59 ± 1.00^b^	8.88 ± 0.58^e^	34.89 ± 1.00^d^	22.51 ± 3.27^cd^
CHI1.25–GO2.0	34.00 ± 0.46^e^	3.50 ± 0.30^cd^	5.17 ± 0.35^f^	64.79 ± 0.47^b^	6.24 ± 0.46^f^	33.71 ± 0.47^d^	21.72 ± 1.60^e^
CH1.5–GO0	50.43 ± 0.45^b^	5.50 ± 0.53^b^	13.63 ± 1.02^b^	50.18 ± 0.66^e^	14.71 ± 0.87^b^	48.29 ± 0.66^a^	38.63 ± 3.17^bc^
CH1.5–GO0.5	44.80 ± 1.40^c^	3.27 ± 0.32^cde^	11.97 ± 0.91^bc^	55.04 ± 1.17^d^	12.41 ± 0.95^c^	43.41 ± 1.17^b^	38.13 ± 1.78^bc^
CH1.5–GO1.0	44.97 ± 0.32^c^	2.33 ± 0.40^def^	10.47 ± 0.42^cd^	54.52 ± 0.23^d^	10.73 ± 0.44^cd^	43.92 ± 0.23^b^	33.25 ± 1.09^cd^
CH1.5–GO1.5	34.90 ± 1.14^e^	2.10 ± 0.17^ef^	9.87 ± 0.32^de^	64.34 ± 1.12^b^	10.09 ± 0.28^de^	34.12 ± 1.11^d^	40.41 ± 1.65^b^
CH1.5–GO2.0	21.33 ± 1.10^f^	1.37 ± 0.32^f^	4.03 ± 0.40^f^	77.27 ± 1.08^a^	4.27 ± 0.39^g^	21.22 ± 1.08^e^	26.96 ± 1.36^de^

***P*-value in ANOVA 2-way**							
CH	0.000	0.000	0.000	0.000	0.000	0.000	0.823
GO	0.000	0.000	0.000	0.000	0.000	0.000	0.000
CH*GO	0.000	0.000	0.000	0.000	0.000	0.000	0.000

Regarding the red/green component (a* values), the addition of GO significantly reduces the red hue of the films compared to the control without GO. Meanwhile, the b* index (yellow/blue component) indicates that CH films with lower GO content tend to exhibit a more pronounced yellow color. The addition of GO causes a noticeable decline in the characteristic yellow tone of chitosan, likely due to the grayish-black color of GO, which diminishes the original yellow-reddish hue of the CH matrix.

The Δ*E* value reflects the overall color difference between the sample and the reference color. As shown in Table 2, the Δ*E* values of films containing GO are higher than those of the control film, and this value increases with increasing GO content. This indicates that the color difference becomes more distinct with higher ratios of GO incorporation. Moreover, at the same GO content, films with a higher CH concentration (1.5%) exhibit greater Δ*E* values than those with a lower concentration (1.25%), suggesting a greater deviation from the reference color.

The color saturation (C*) of the films is also affected by the presence of GO. Films without GO exhibit higher C* values, indicating brighter and more saturated colors. As the GO content increases, the C* value exhibits a decreasing trend, particularly at the 2.0% level, indicating that the films become darker and less vibrant. Furthermore, CH films exhibiting higher polymer concentration (1.5%) exhibit lower C* values than those with a lower CH concentration (1.25%), indicating that a denser CH matrix yields darker, less vivid films.

The whiteness index (WI) of the film without GO shows the highest value, reflecting the natural white color of pure chitosan. With the addition of GO, the WI decreases progressively, due to the dark coloration of GO particles, which reduces the whiteness and shifts the film color toward gray or dark brown tones.

Similarly, the yellowness index (YI) of the control film is the highest, indicating the characteristic yellow hue of chitosan. Upon adding GO, the YI drops sharply, especially at contents from 1.0% and above, where the yellow color was almost completely masked, shifting to a gray appearance.

The results of the two-way ANOVA analysis presented in Table 2 showed that variations in CH concentration and the amount of GO added, as well as the interaction between CH and GO, had statistically significant effects (*p* < 0.05) on all color parameters (L*, a*, b*, Δ*E*, C*, and WI) of the obtained films. Meanwhile, CH concentration did not significantly affect the YI of the films (*p* = 0.823 > 0.05), whereas both the GO content and the CH × GO interaction had significant effects on the YI values of the films.

When comparing different CH concentrations, it was observed that higher CH concentrations produced darker films with lower color saturation. In contrast, increasing the proportion of GO led to darker films due to its strong light absorption characteristics. Moreover, the redness and yellowness of films containing higher GO levels decreased markedly, with the film color gradually shifting toward the dark gray of GO. The interaction between CH and GO was statistically significant (*p* < 0.05), indicating that the effect of GO depended on the CH concentration. At higher CH concentrations (1.5%), the darkening effect of GO was more pronounced compared to films prepared with 1.25% CH. This may be attributed to the higher viscosity of the chitosan solution, which promotes GO particle aggregation, enhancing light absorption and reducing film brightness. Additionally, the formation of hydrogen bonds between the hydroxyl and amino groups of chitosan and the oxygen-containing functional groups of GO may alter the microstructure and light reflectance of the films, thereby significantly affecting their color characteristics.

The change in color of GO-containing films can be explained by the strong light absorption capacity and the inherently dark color of GO, which masks the natural color of pure CH films when dispersed into the polymer matrix. The alteration in the color properties of chitosan–GO films not only reflects the influence of GO on the optical characteristics of the packaging material but also has implications for consumer acceptance. Generally, lighter and more transparent films are preferred for fresh fruit packaging because they allow consumers to assess the natural color and freshness of the product visually. In contrast, darker films with higher GO contents may reduce visual appeal by obscuring the fruit color, potentially decreasing consumer willingness to purchase. However, such darker films could be advantageous for packaging products that require protection from light-induced degradation, where visual transparency is less critical. Therefore, the application of CH–GO films largely depends on the properties of the specific food product to be packaged, to select an appropriate GO content. In other words, both the functional preservation properties and the aesthetic compatibility with the appearance of the packaged food should be considered to determine the optimal GO concentration in the chitosan matrix.

### 3.6. Optical properties

The optical properties of materials could significantly influence their potential applications, particularly in food preservation. The optical characteristics of CH–GO films were evaluated and were presented in Table 3.

**Table 3:** Optical properties of CH–GO films

Sample	Light transmittance (%) at wavelengths								Opacity, AU per mm
	350 nm	400 nm	450 nm	500 nm	550 nm	600 nm	650 nm	700 nm	
CH1.25–GO0	1.03 ± 0.06^ab^	5.10 ± 0.10^a^	20.43 ± 2.33^ab^	43.63 ± 2.33^a^	62.10 ± 2.08^a^	66.97 ± 1.97^a^	75.07 ± 4.16^a^	78.57 ± 1.17^a^	0.12 ± 0.01^d^
CH1.25–GO0.5	0.97 ± 0.06^ab^	2.93 ± 0.06^e^	16.37 ± 0.06^cd^	34.03 ± 3.09^b^	47.07 ± 3.35^bc^	47.40 ± 1.41^c^	51.30 ± 2.18^bc^	51.90 ± 1.74^bc^	0.32 ± 0.01^ab^
CH1.25–GO1.0	0.67 ± 0.06^cde^	2.00 ± 0.20^f^	15.07 ± 1.31^cde^	30.30 ± 1.71^b^	44.37 ± 1.25^bc^	44.17 ± 0.60^c^	49.87 ± 2.12^c^	47.57 ± 1.08^c^	0.32 ± 0.01^ab^
CH1.25–GO1.5	0.60 ± 0.10^de^	1.50 ± 0.00^g^	11.87 ± 0.81e^f^	21.70 ± 0.35^c^	32.83 ± 4.09^d^	34.57 ± 2.55^d^	35.17 ± 4.37^d^	34.07 ± 1.34^d^	0.28 ± 0.02^b^
CH1.25–GO2.0	0.53 ± 0.06^e^	1.20 ± 0.00^h^	10.70 ± 0.17^f^	20.93 ± 1.33^c^	27.90 ± 2.05^d^	26.63 ± 1.29^e^	27.77 ± 1.93^d^	30.23 ± 4.51^d^	0.29 ± 0.01^b^
CH1.5–GO0	1.17 ± 0.15^a^	5.30 ± 0.00^a^	22.80 ± 0.26^a^	46.87 ± 2.80^a^	60.73 ± 3.27^a^	61.93 ± 2.21^a^	69.77 ± 3.35^a^	79.30 ± 1.51^a^	0.13 ± 0.01^d^
CH1.5–GO0.5	1.00 ± 0.00^ab^	4.40 ± 0.00^b^	17.53 ± 2.83^bc^	41.53 ± 3.04^a^	51.43 ± 0.23^b^	54.77 ± 3.02^b^	59.30 ± 4.96^b^	57.43 ± 3.56^b^	0.22 ± 0.02^c^
CH1.5–GO1.0	0.93 ± 0.12^abc^	3.47 ± 0.06^c^	15.50 ± 0.50^cd^	34.27 ± 1.68^b^	48.30 ± 2.25^bc^	49.63 ± 2.22^bc^	54.77 ± 3.15^bc^	51.30 ± 3.81^bc^	0.28 ± 0.01^c^
CH1.5–GO1.5	0.83 ± 0.15^bcd^	3.20 ± 0.00^d^	13.40 ± 1.31^def^	31.30 ± 0.46^b^	43.47 ± 3.21^c^	45.27 ± 2.11^c^	49.70 ± 0.14^bc^	48.73 ± 1.80^c^	0.20 ± 0.01^c^
CH1.5–GO2.0	0.50 ± 0.10^e^	1.60 ± 0.00^g^	5.97 ± 0.93^g^	13.60 ± 2.33^d^	18.07 ± 2.01^e^	20.17 ± 2.53^f^	17.93 ± 1.82^e^	16.65 ± 1.91^e^	0.35 ± 0.03^a^

***P*-value in ANOVA 2-way**									
CH	0.002	0.000	0.722	0.000	0.120	0.005	0.044	0.025	0.000
GO	0.000	0.000	0.000	0.000	0.000	0.000	0.000	0.000	0.000
CH*GO	0.065	0.000	0.000	0.000	0.000	0.000	0.000	0.000	0.000

The results showed that the light transmittance of the films gradually increased with increasing wavelength, indicating that the films effectively blocked near-ultraviolet (UV-A) radiation (wavelengths ranging from 315 to 400 nm), while still allowing better transmission of visible light in the yellow-to-red region. This property is highly beneficial for food packaging, as the film can block UV radiation that causes oxidation and deterioration of product quality. These observations were consistent with previous studies by Han *et al.*, which also reported increased transmittance of CH–GO films with increasing wavelength.^28,42^ Additionally, the data in Table 3 indicate that increasing the GO content in the films results in a general decrease in light transmittance at both CH concentrations. This was attributed to the layered structure and strong light absorption capacity of GO, which obstructed the path of light when dispersed in the CH matrix, thus significantly reducing the material's optical transmittance. Similar trends had been previously reported for 1.5% and 2.0% CH films,^28^ 4.0% CH films,^42^ PLA-based films,^45^ and furcellaran-based nanocomposite films.^46^

Notably, Table 3 also showed that increasing the CH concentration from 1.25% to 1.5% slightly improved the light transmittance of the films. However, for films containing 2% GO, the transmittance of the 1.5% CH film was lower than that of the 1.25% film. This suggests that at high GO concentrations, poor dispersion and GO aggregation may occur, enhancing light absorption and scattering, which significantly reduces the film's transparency.

The two-way ANOVA analysis showed that the effect of GO content on light transmittance was highly significant (*p* < 0.05) at all wavelengths, whereas the CH concentration only affected transmittance at certain wavelengths. Meanwhile, the interaction between CH and GO also had a notable impact on the optical transmittance of the films, indicating that the influence of GO on the optical properties of the films depended on the CH concentration in the film-forming formulation.

Exposure to UV radiation was known to accelerate the oxidation of food components.^46^ Therefore, the strong UV-blocking ability of GO-containing films suggested their potential application in packaging and preserving light-sensitive food products.

The results in Table 3 also show that CH films without GO exhibited relatively low opacity, or in other words, high transparency. When GO was added up to 1.0%, the opacity of the films increased significantly; however, further increasing the GO content beyond 1.0% led to a slight decrease in opacity. Meanwhile, opacity in films containing 1.5% CH increased proportionally with the GO content. Similar observations were previously reported for CH films containing 1.5% and 2.0% CH with added GO^28^ and for films containing 4.0% CH.^42^ Conversely, Han *et al.* reported that increasing the amount of GO resulted in reduced light transmittance of the films.^38^

This apparent inconsistency among studies could be attributed to several factors, including differences in the dispersion state of GO within the polymer matrix, the synthesis and casting methods employed, and the intrinsic optical behavior of the nanomaterials used. GO sheets can disperse uniformly at low GO concentrations, enhancing light scattering and increasing opacity. However, at higher concentrations, aggregation of GO sheets may occur, forming microdomains that can either reflect or transmit light depending on their orientation and packing density, thus reducing the overall opacity slightly. Additionally, the viscosity of the film-forming solution and the degree of interaction between CH and GO are essential in determining optical clarity. A higher CH concentration increases the matrix density and limits the mobility of GO sheets, which can lead to a more homogeneous structure but higher light scattering. Overall, these results suggest that the optical properties of CH–GO films result from a delicate balance between GO content, CH concentration, and dispersion uniformity, which together determine the visual appearance and potential packaging applications of the films.

The incorporation of GO enhances the UV-blocking capability of the films while maintaining an appropriate level of light transmittance in the visible region and adjusting film opacity according to application requirements. Therefore, CH–GO films hold great potential for use in packaging light-sensitive food products such as edible oils, milk and dairy products, fruit juices, vitamin-rich foods (particularly those containing vitamins A, B_2_, and C), or products with natural pigments such as carotenoids and anthocyanins. UV-blocking films can help reduce lipid oxidation, vitamin degradation, and color changes, thereby extending shelf life and preserving the sensory quality of foods.

### 3.7. Water absorption capacity, solubility, and water vapor permeability

**Fig. 6:**
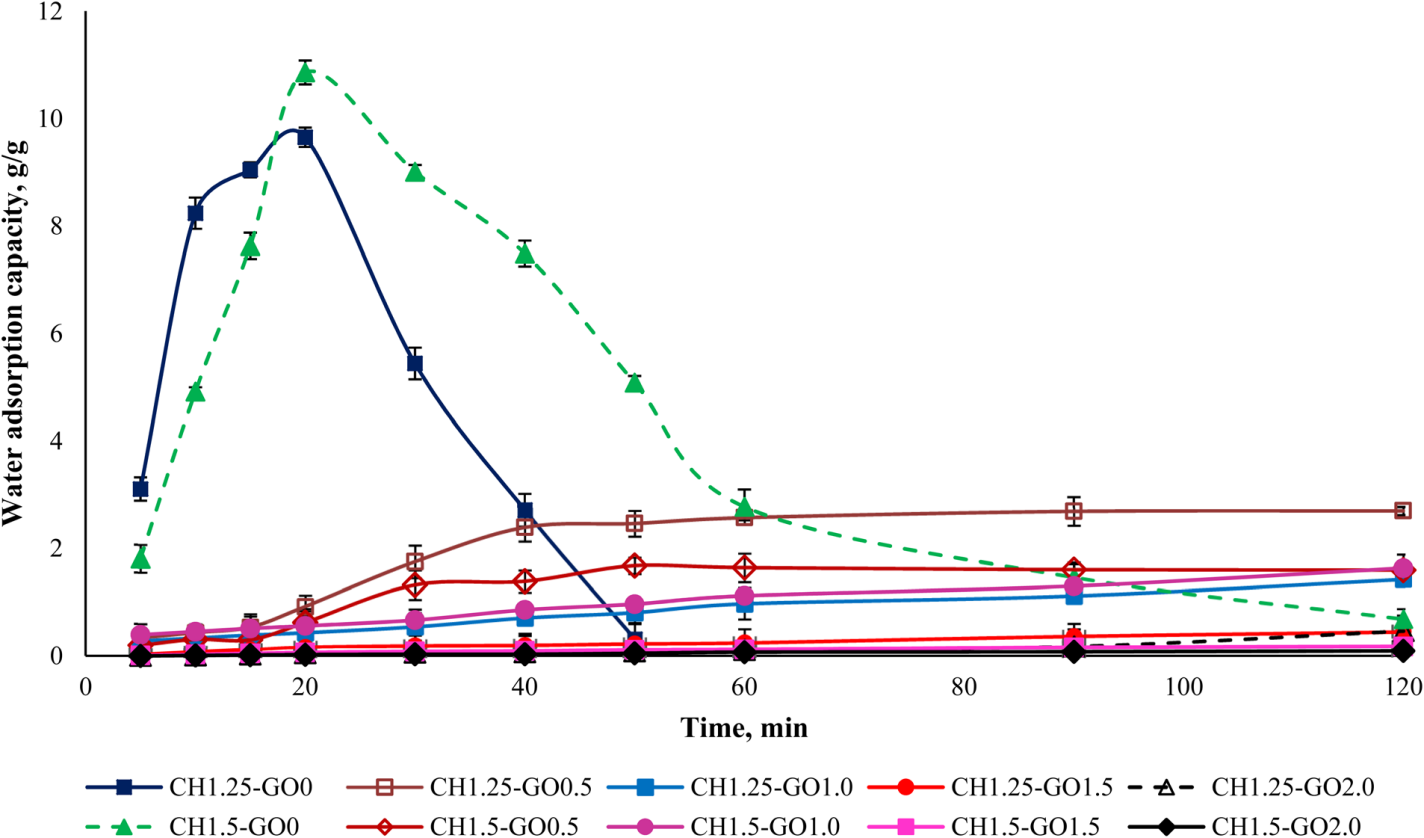
Water absorption capacity of CH–GO films.

The water absorption capacity of the studied films was illustrated in Fig. 6. The data indicated that CH films possessed a relatively high-water absorption ability, reaching the maximum uptake after approximately 20 minutes. After this point, the films began to dissolve gradually. Films with a lower CH ratio dissolved faster than those with a higher CH content. This may be due to a less compact polymer network structure, resulting in faster water absorption and dispersion. When GO was incorporated into the films, the water absorption capacity decreased significantly, and the water uptake decreased with increasing GO content. Notably, the water absorption capacity of films containing 1.5–2.0% GO was found to be very low.

The reduction in water uptake observed in CH–GO films was attributed to the formation of interactions between GO molecules and the hydrophilic functional groups in chitosan, such as hydroxyl and amine groups. These interactions reduced the number of available hydrophilic sites, thus limiting the ability to form hydrogen bonds with water molecules. Moreover, the layered, porous, and rough structure of GO further obstructed direct contact between water and the polymer matrix, acting as a physical barrier that hindered water absorption and resulted in a notable decrease in the material's water uptake.

Thus, the incorporation of GO into CH significantly reduces the water absorption capacity of the material. The decrease in hydrophilicity enhances the film's stability in humid environments while limiting its swelling and dissolution under high-moisture conditions such as in food storage. With these characteristics, the CH–GO films are suitable for practical applications such as food packaging, fruit coating, or pharmaceutical coatings, where good water vapor resistance and biocompatibility are required.

The solubility of a material significantly affects its properties and potential applications in various industrial fields; therefore, it is an essential factor to investigate. The solubility results of the films presented in Table 4 show that CH films incorporated with GO exhibited slightly lower solubility compared to the pure CH film. However, as more GO was added to CH, the solubility decreased. ANOVA revealed that the CH ratio and the interaction between CH and GO did not have a statistically significant effect on film solubility. Nevertheless, the variation in GO content was identified as the main factor influencing the solubility of CH–GO films. This phenomenon can be explained by the fact that although GO is dispersible and hydrophilic due to the presence of numerous polar functional groups, such as –OH, –COOH, and C=O in its chemical structure, which promote strong interactions with water, these groups can also form hydrogen or electrostatic bonds with hydroxyl and amine groups in chitosan when incorporated into the chitosan matrix. As a result, the water solubility of the CH–GO film decreases compared to the pure CH film. The solubility of the CH–GO films in this study was considerably higher than that reported by Han Lyn,^28^ which may be attributed to differences in the drying conditions. In this study, the materials were dried at 60 °C, whereas Han Lyn's films were air-dried. Drying at a higher temperature could weaken the intermolecular bonding network, thereby increasing solubility. In addition, variations in the source of chitosan may significantly affect the materials' solubility. Overall, although the reduction in solubility was not substantial, the addition of an appropriate amount of GO could still improve the water resistance of CH films, making the material more suitable for applications requiring high stability in humid environments, such as food packaging or edible coatings for fresh fruits.

**Table 4:** Solubility, Water vapor permeability and Mechanical properties of CH–GO films

Sample	Solubility, %	Water vapor permeability	Tensile strength, MPa	Elongation at break, %	Antioxidant activity against DPPH radicals, %	Antibacterial activities, mm	
						*E. coli*	*S. aureus*
GO	—	—	—	—	—	55.12 ± 1.43^a^	21.58 ± 0.78^a^
CH1.25–GO0	66.83 ± 1.26^a^	6.69 ± 0.21^a^	16.69 ± 0.09^a^	4.57 ± 0.09^fg^	37.76 ± 3.73^b^	24.37 ± 1.38^bc^	11.67 ± 0.57^a^
CH1.25–GO0.5	63.11 ± 1.89^a^	5.38 ± 0.17^bc^	17.17 ± 0.13^a^	4.85 ± 0.10^ef^	36.03 ± 0.75^b^	23.80 ± 1.28^c^	12.43 ± 0.81^a^
CH1.25–GO1.0	61.88 ± 2.28^a^	4.42 ± 0.12^de^	13.13 ± 0.61^b^	5.50 ± 0.15^d^	14.90 ± 3.79^d^	24.13 ± 4.23^c^	12.03 ± 0.75^a^
CH1.25–GO1.5	62.52 ± 0.83^a^	4.08 ± 0.34^efg^	5.85 ± 0.47^ef^	2.74 ± 0.25^h^	10.38 ± 0.18^d^	26.27 ± 2.72^bc^	14.53 ± 1.60^a^
CH1.25–GO2.0	61.31 ± 4.41^a^	3.54 ± 0.25^fgh^	5.05 ± 0.15^f^	2.79 ± 0.18^h^	10.45 ± 1.98^d^	25.90 ± 1.71^bc^	12.93 ± 0.32^a^
CH1.5–GO0	64.82 ± 1.16^a^	6.07 ± 0.52^ab^	5.32 ± 0.41^f^	4.20 ± 0.22^g^	58.17 ± 5.64^a^	26.70 ± 3.18^bc^	11.43 ± 0.38^a^
CH1.5–GO0.5	61.78 ± 2.69^a^	4.83 ± 0.05^cd^	7.12 ± 0.13^cd^	9.09 ± 0.07^c^	36.46 ± 1.83^b^	27.50 ± 5.90^bc^	11.07 ± 0.87^a^
CH1.5–GO1.0	61.02 ± 1.65^a^	4.28 ± 0.20^def^	7.51 ± 0.16^c^	10.62 ± 0.12^a^	24.62 ± 1.02^c^	33.03 ± 2.62 ^ab^	13.67 ± 0.47^a^
CH1.5–GO1.5	61.49 ± 1.95^a^	3.49 ± 0.25^gh^	6.37 ± 0.30^de^	9.53 ± 0.10^b^	15.57 ± 1.59^d^	36.27 ± 1.50^a^	13.60 ± 2.49^a^
CH1.5–GO2.0	62.25 ± 2.66^a^	3.00 ± 0.16^h^	3.91 ± 0.35^g^	5.19 ± 0.07^de^	10.72 ± 1.85^d^	36.37 ± 2.21^a^	13.23 ± 1.99^a^

***P*-value in ANOVA 2-way**							
CH	0.308	0.000	0.000	0.000	0.000	0.000	0.793
GO	0.019	0.000	0.000	0.000	0.000	0.005	0.013
CH*GO	0.838	0.562	0.000	0.000	0.000	0.085	0.293

The water vapor permeability (WVP) of the films also demonstrated a decreasing trend upon the addition of GO into the chitosan matrix. A higher GO content corresponded to lower water vapor permeability. CH films with a lower polymer concentration (1.25%) formed looser and weaker polymer networks, allowing water vapor molecules to diffuse more easily compared to films with a higher CH concentration. The porous, multilayered structure and rough surface of GO contributed to the formation of a mechanical barrier that hindered water vapor movement and diffusion through the films, thus reducing the WVP of the material.

The water vapor permeability (WVP) of the studied films showed that incorporating GO into CH films led to a gradual decrease in WVP. The higher the GO content, the lower the water vapor permeability. CH films with a lower CH concentration (1.25%) formed a looser, weaker polymer network, allowing water molecules to pass through more easily than in films with a higher CH content. When GO was incorporated into CH, its porous, multilayered structure created a rough, uneven surface with many ridges and peaks, hindering the movement and diffusion of water vapor across the film and thereby reducing its permeability.

This result is consistent with the findings of Vilvert *et al.*,^30^ who reported that the addition of GO significantly reduced the WVP of pure CH films. However, in this study, the WVP of the CH–GO films decreased by 47.09–50.58% compared to the control film, a reduction higher than that reported by Vilvert *et al.* Two-way ANOVA revealed that both CH and GO factors had statistically significant effects on the material's WVP, whereas the CH × GO interaction was not significant (*p* = 0.562 > 0.05). This indicates that the impact of GO on WVP was similar at both CH ratios investigated. Low water vapor permeability is a crucial factor determining the effectiveness of biopolymer films in food packaging applications. Films with reduced WVP help minimize product moisture loss and prevent environmental water vapor from penetrating the product, thereby maintaining the structure, weight, and sensory quality of foods during storage. Therefore, the significant improvement in water vapor barrier properties of CH films upon GO incorporation demonstrates their high potential for packaging applications involving fresh, moisture-sensitive, or easily dehydrated products such as fruits, vegetables, meat, fish, or dried goods. In addition, this property enhances the mechanical strength and stability of the film, enabling CH–GO films to replace conventional plastic materials in environmentally friendly packaging applications partially.

### 3.8. Mechanical properties

The tensile strength and elongation at break of the films were presented in Table 4. The results indicated that both the CH concentration and the GO content significantly affected the tensile strength of the material with statistical significance level *p* < 0.05. When GO was incorporated into the CH films, the tensile strength initially increased and then decreased at both CH concentrations. Specifically, for the films with a lower CH content (1.25%), the addition of 0.5% GO slightly improved the tensile strength. However, when the GO content increased further, the tensile strength decreased markedly. In contrast, for films with a higher CH content (1.5%), the addition of 1.0% GO significantly enhanced the tensile strength. However, further increases beyond this ratio resulted in a decline in tensile strength. This trend was attributed to the fact that, at low concentrations, GO particles dispersed well and interacted effectively with the chitosan matrix, thus reinforcing the polymer network. However, at higher concentrations, GO particles tended to agglomerate, disrupting the polymer structure and weakening the film's tensile strength.

Therefore, to develop packaging films with high mechanical strength, incorporating GO at low ratios (approximately 0.5–1.0%) was considered suitable. Additionally, films with 1.25% CH showed higher tensile strength than those with 1.5%, possibly due to differences in polymer density and network structure.

It was also reported that CH films with 2.0% CH exhibited higher tensile strength than those with 1.5%, according to a study by Lyn and *et al.*^28^ Nevertheless, similar to this study, Lyn and *et al.* also observed that increasing GO content reduced tensile strength. Such differences could be attributed to the intrinsic properties of the chitosan used or the drying technique applied in film preparation. In this study, the films were oven-dried at 50 °C, whereas the previous study used natural drying. The higher drying temperature might have disrupted the polymer network and weakened the molecular interactions, resulting in lower mechanical strength. However, the results in this study aligned with the findings of Gea *et al.* regarding CH–GO composite films.^39^

Regarding elongation at break (also shown in Table 4), the CH and GO ratios influenced the elasticity of the films. For the 1.25% CH group, the addition of GO from 0.5% to 1.0% increased elongation but further increases beyond 1.0% significantly reduced this property. For the 1.5% CH group, all films containing GO exhibited greater elongation than the control film without GO. Similar to the 1.25% group, adding up to 1.0% GO improved elongation, but excess GO led to a gradual decline. This reduction was explained by the aggregation of GO at high concentrations, which disrupted the uniformity of the polymer matrix and weakened its flexibility and elasticity. Moreover, films with a higher CH content (1.5%) demonstrated better elongation than those with 1.25%, suggesting that increased polymer density enhanced film flexibility and stretchability.

In conclusion, the results indicated that the addition of GO at appropriate levels could significantly enhance both the tensile strength and elongation of the films, particularly in the 1.5% CH matrix. Overall, the 1.5% CH film supplemented with 1.0% has the most improved tensile strength (increased by 1.41 times) and elongation (increased by 2.53 times) compared to the original sample and samples with the same content, making it a promising candidate for applications in flexible packaging, biomedical materials, or functional coatings.

### 3.9. Antioxidant and antibacterial activities

Antioxidant and antibacterial activities were two important properties considered when selecting materials for food packaging and preservation applications. The results of DPPH free radical scavenging activity and antibacterial efficacy against *E.coli* and *S. aureus* of the CH–GO films were presented in Table 4.

The data showed that, for both CH concentrations, the antioxidant capacity of the films gradually decreased as the GO content increased. However, at a low GO content (0.5%), the free radical scavenging ability remained comparable to that of the pure CH film. From 1.0% GO onward, the DPPH scavenging efficiency significantly declined. When comparing the two CH concentrations, the films containing a higher CH ratio (1.5%) exhibited better radical scavenging activity than those with 1.25% chitosan. This was likely attributed to the higher abundance of functional groups such as –OH and –NH_2_ in chitosan, which could interact with and neutralize free radicals. Nevertheless, when GO was incorporated, its particles tended to interact with these functional groups, thereby reducing the number of available reactive sites and diminishing the overall antioxidant activity. The decrease in antioxidant activity with increasing GO content may result from interactions between the oxygen-containing functional groups of GO (such as –COOH, –OH, and epoxy) and the amino and hydroxyl groups of chitosan. These interactions reduce the number of free functional groups available to react with DPPH radicals, thereby lowering the radical-scavenging efficiency. Additionally, SEM observations (Fig. 3) revealed that at low GO concentrations, GO sheets were relatively well-dispersed within the polymer matrix, allowing DPPH radicals to diffuse and interact with chitosan. However, as the GO content increased (≥1.0%), the GO sheets tended to cover the film surface, hindering DPPH diffusion and reducing its contact with the chitosan active sites. Both factors contributed to the significant decline in the antioxidant activity of the material with increasing GO content.

Regarding antibacterial activity, all film samples exhibited inhibitory effects against both *E. coli* and *S. aureus*, with greater inhibition observed against *E. coli.* As the GO content increased, the antibacterial effectiveness against *E. coli* also improved, especially in films containing 1.5% CH and 1.5–2.0% GO, which could increase the diameter of the inhibition zone for the *E. coli* strain by 26.66–35.84% and for the *S. aureus* strain by 13.24–18.99% compared to the original film. In contrast, changes in GO content had little effect on the inhibition of *S. aureus*, with most samples demonstrating comparable antibacterial activity. This difference was attributed to variations in bacterial cell wall structures: Gram-negative bacteria (*E. coli*) possessed thinner cell walls than Gram-positive bacteria (*S. aureus*), making them more vulnerable to disruption by the film surface. GO molecules, with their rough surfaces and sharp edges, can cause damage to bacterial cell membranes upon direct contact, resulting in a pronounced antibacterial effect.^47,48^ Therefore, incorporating GO into CH films produces surfaces with relatively high roughness, sharp-edged structures, and dense GO coverage, thereby enabling more effective rupture of bacterial cell membranes. In addition, previous studies have shown that GO can damage bacterial membranes by inducing oxidative stress within cells, thereby exerting antibacterial effects upon direct contact.^49,50^ Due to the inherent antibacterial properties of GO, increasing its concentration in CH films further enhances the antibacterial activity of the resulting composite films.

From the results, it can be observed that CH–GO films may not be the optimal choice for packaging or preserving foods that are highly susceptible to oxidation, such as butter, meat, or high-fat fruits. This is because GO can reduce the film's antioxidant activity, making it less effective at inhibiting lipid oxidation in fat-rich products. In contrast, CH–GO films appear more suitable for preserving fresh fruits or low-fat foods, where lipid oxidation is not the main factor in quality degradation. In particular, for dark-colored fruits such as plums, grapes, or strawberries, the antimicrobial and water-retention capabilities of CH–GO films can help maintain freshness and natural color during storage.

For plums – fruits with high water content that are prone to wrinkling and losing freshness after harvest – the CH–GO films, with their low water vapor permeability and solubility, can effectively minimize moisture loss and reduce weight shrinkage during storage. Furthermore, the improved tensile strength and elongation at suitable GO contents enhance the film's mechanical resistance during packaging, transportation, and stacking, preventing tearing or cracking. Consequently, the film adheres well to the fruit surface, forming a uniform protective coating that maintains natural gloss and reduces discoloration and surface browning. Notably, plums are rich in anthocyanins, compounds that are highly sensitive and easily degraded under unsuitable storage conditions, particularly when exposed to UV and short-wavelength visible light (350–450 nm). Upon illumination, anthocyanins may undergo photooxidation, leading to color fading, decreased antioxidant activity, and reduced sensory quality. Therefore, CH–GO films with reduced light transmittance are advantageous for preserving anthocyanin-rich fruits, as they can limit pigment degradation during storage.

Overall, with their excellent moisture barrier properties, high mechanical strength, suitable optical characteristics, and strong antibacterial activity, CH–GO films show great potential as effective packaging materials for plums. They can help maintain color and nutritional value and extend shelf life. Moreover, these materials offer promising applications in biodegradable packaging, partially replacing conventional plastic films and contributing to eco-friendly and sustainable preservation solutions for naturally pigmented fruits such as plums, mulberries, blueberries, and cherries.

### 3.10. Preservation ability of plums by the studied films

#### 3.10.1. Visual observation of plums during storage

Visual observations of the control and plums preserved using the studied films, before and during storage, are presented in Table 5. Initial observations revealed that all fruits were fresh before storage, characterized by tight skins, non-glossy surfaces, and no visible signs of deterioration. After coating the films, the fruit surfaces became glossier and appeared fresher. By day 2, the control sample began to show slight discoloration, with the skin becoming darker, while the coated fruits remained fresh, glossy, and nearly unchanged in surface appearance. Notably, the samples preserved with CH1.25–GO2.0 and CH1.5–GO2.0 films maintained delicious freshness. By day 4, the control and the fruits coated with the 1.25% CH film began to exhibit surface deterioration, while the other samples still retained their gloss, color, and freshness. By day 6, the decline of the 1.25% CH film sample had become more severe. The control sample exhibited significant signs of decay starting from day 8. By day 10, the stem end had rotted, and the fruit surface had visibly lost its gloss since day 6. In contrast, the fruits preserved with 1.5% CH film and CH films containing GO maintained their gloss and freshness until day 10. Most fruits showed slight stem-end spoilage by day 10, except those preserved in CH1.25–GO2.0, CH1.5–GO1.5, and CH1.5–GO2.0 films.

**Table 5:** Visual observations of the control and plums preserved using the studied films during the storage period

Sample		The storage time					
		Day 0	Day 2	Day 4	Day 6	Day 8	Day 10
Plums preserved using the studied films	Control	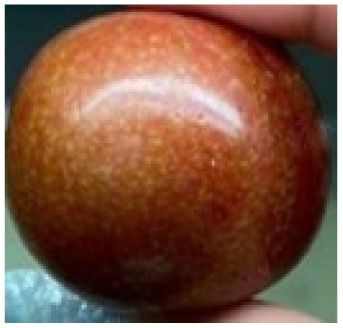	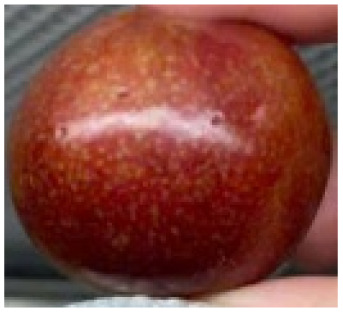	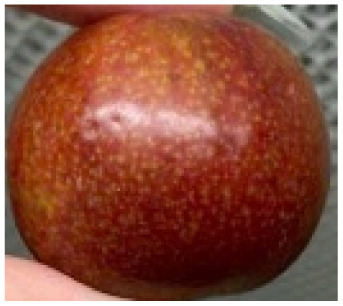	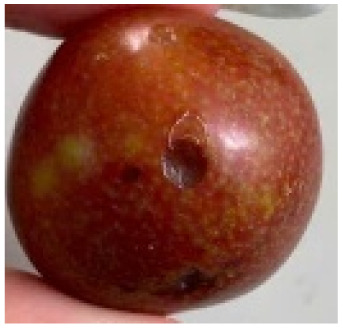	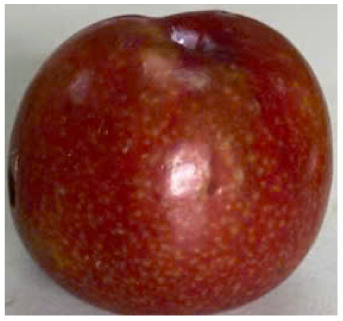	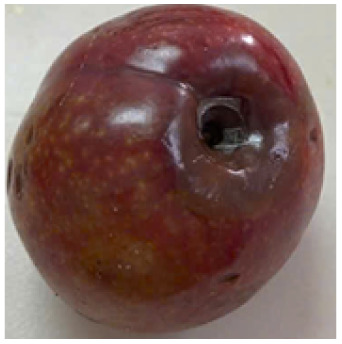
	CH1.25–GO0	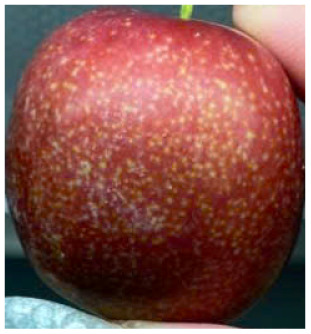	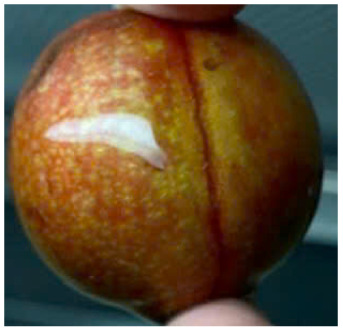	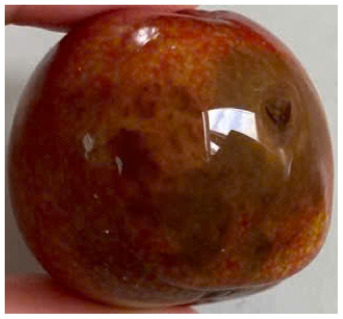	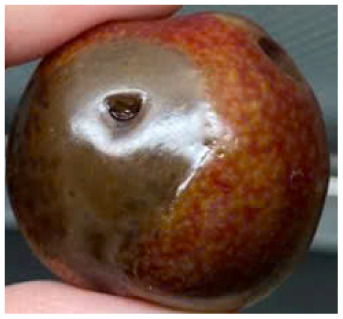	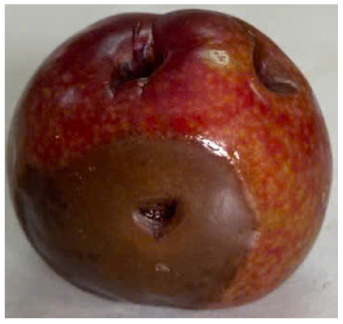	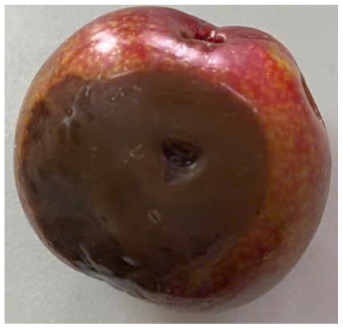
	CH1.25–GO0.5	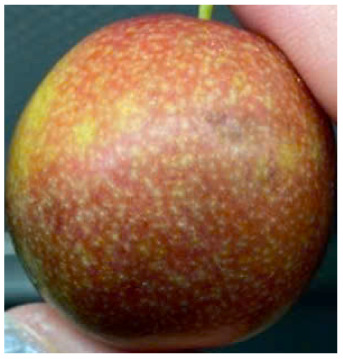	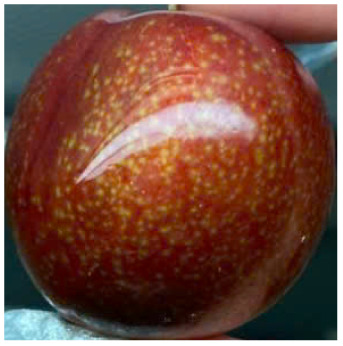	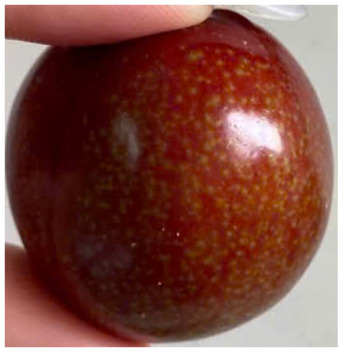	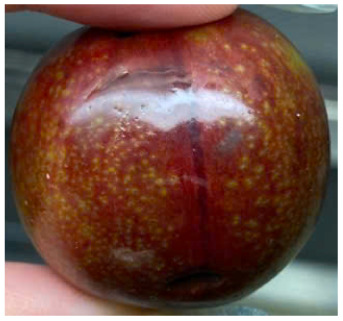	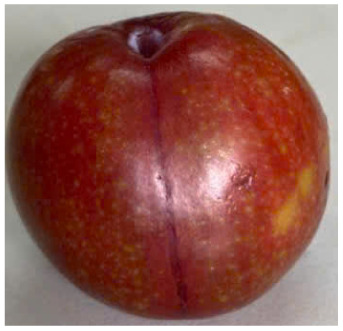	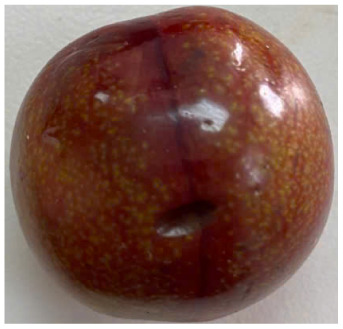
	CH1.25–GO1.0	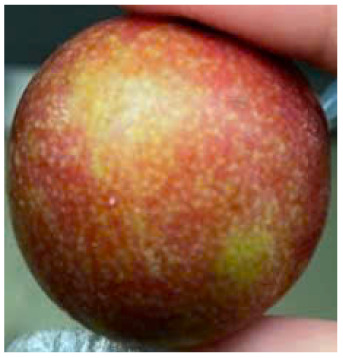	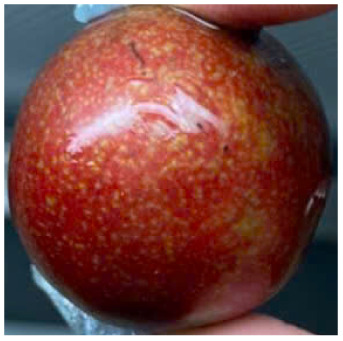	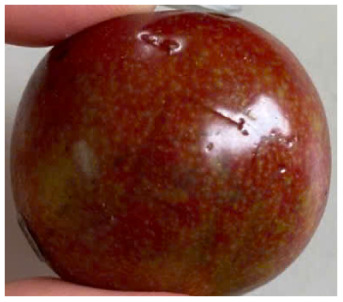	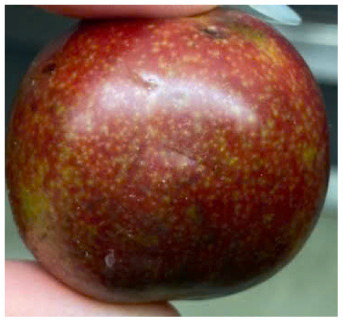	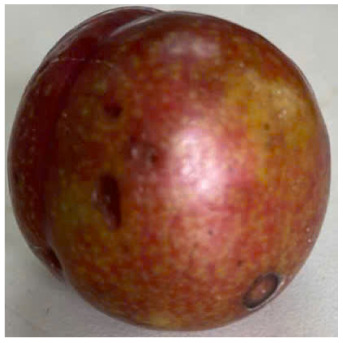	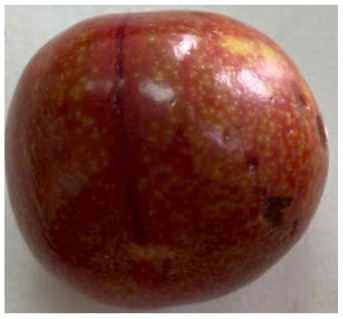
	CH1.25–GO1.5	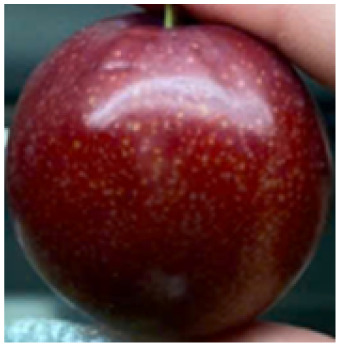	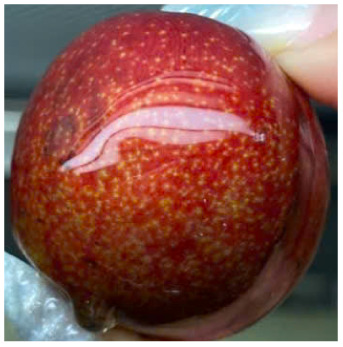	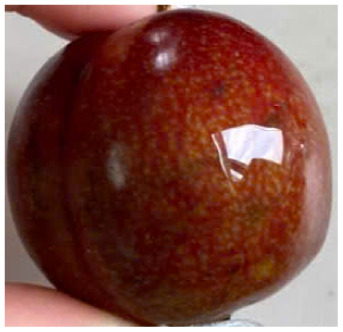	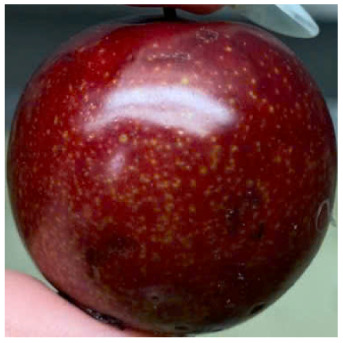	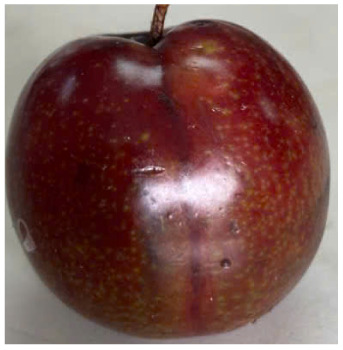	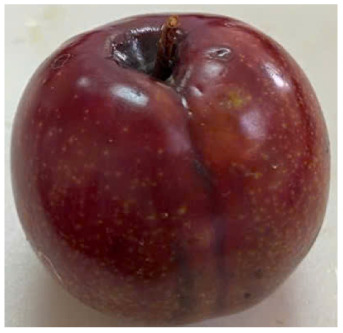
	CH1.25–GO2.0	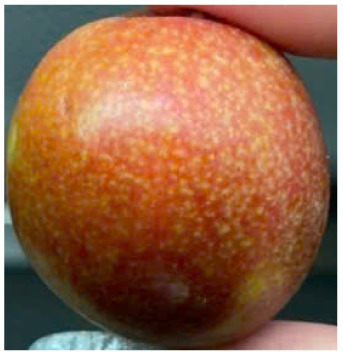	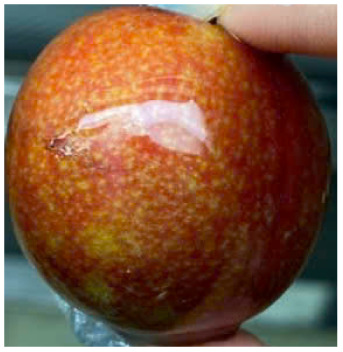	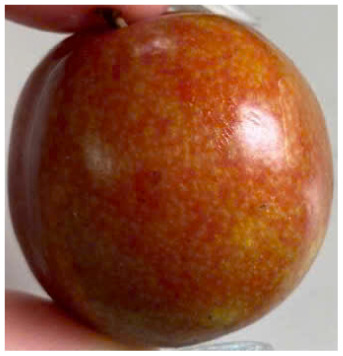	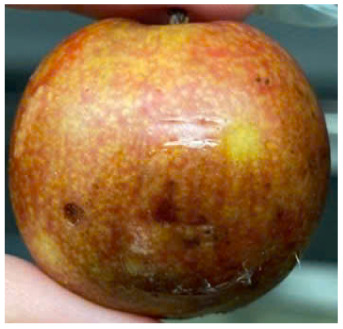	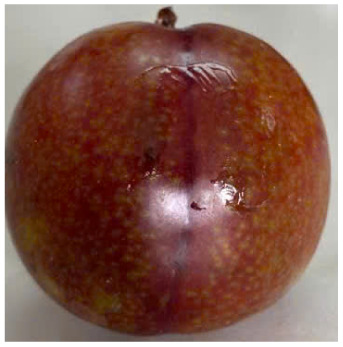	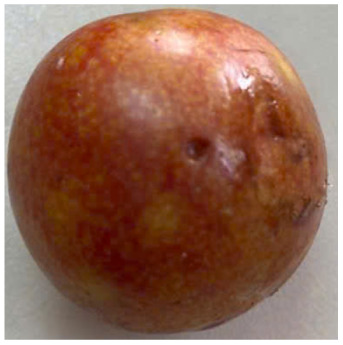
	CH1.5–GO0	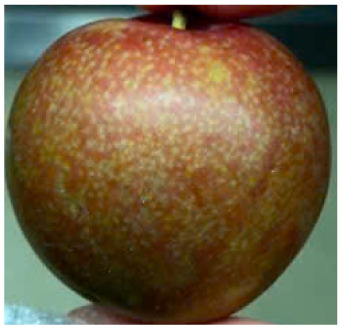	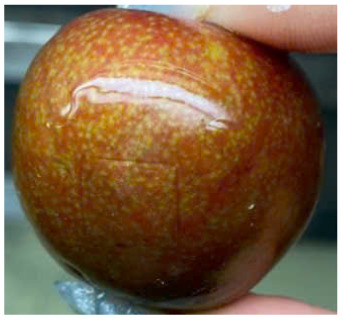	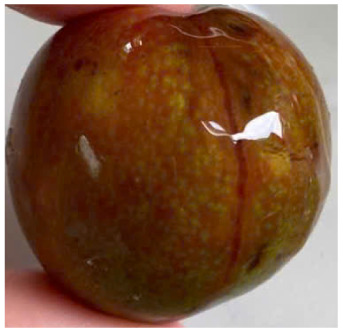	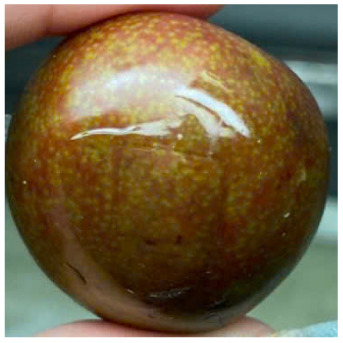	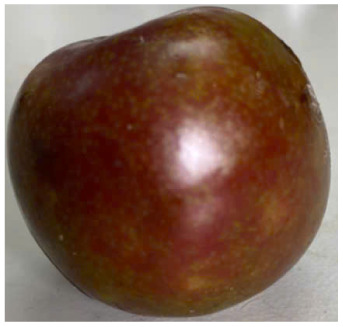	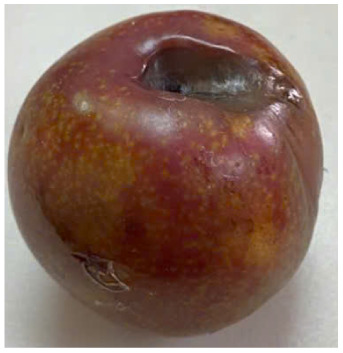
	CH1.5–GO0.5	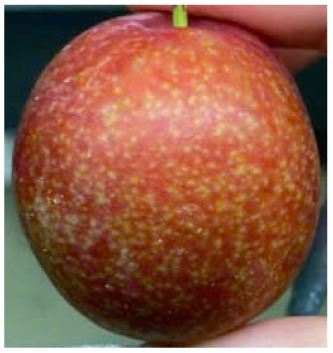	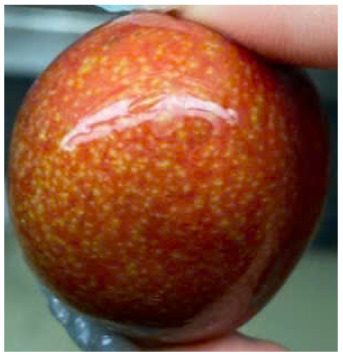	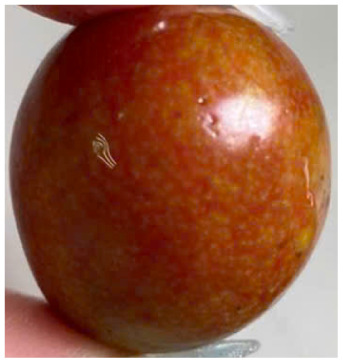	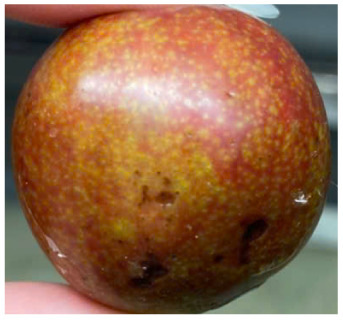	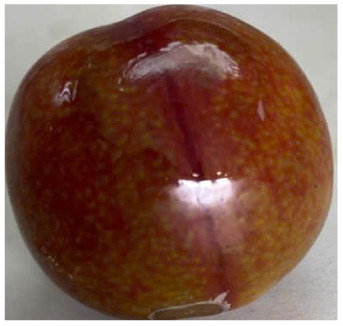	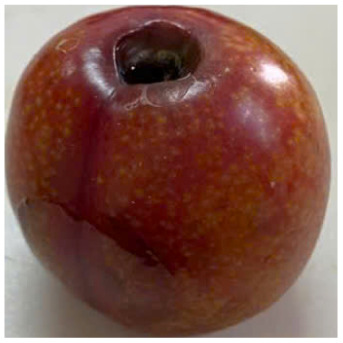
	CH1.5–GO1.0	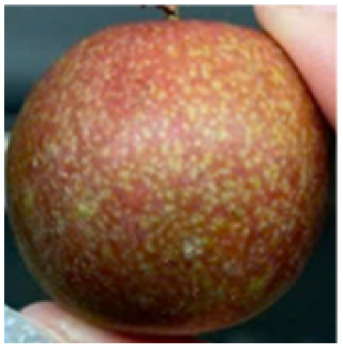	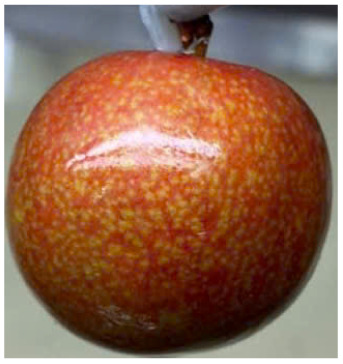	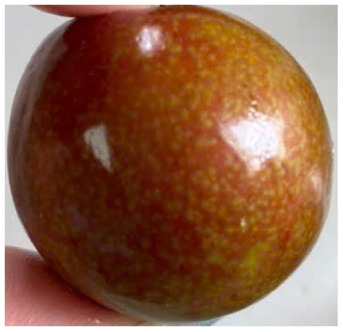	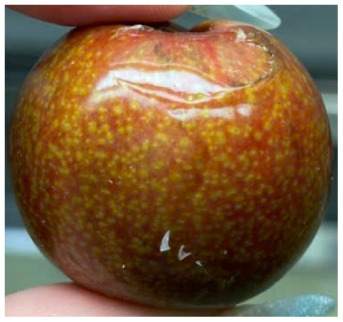	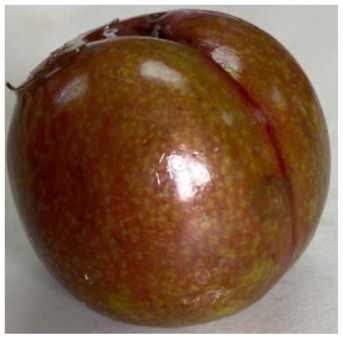	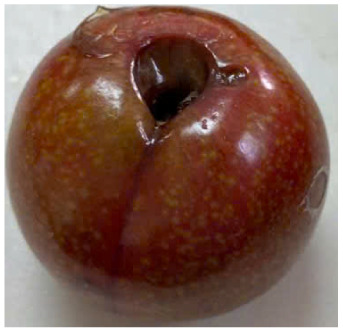
	CH1.5–GO1.5	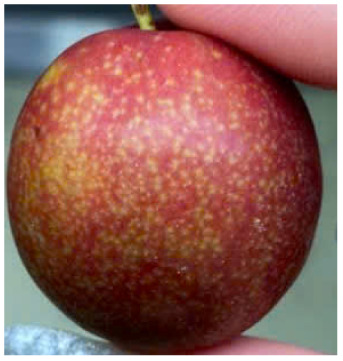	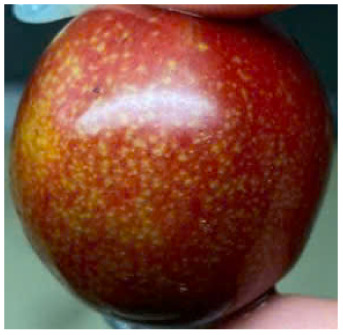	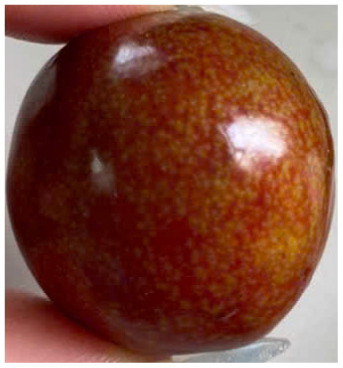	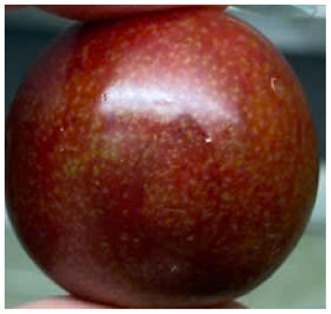	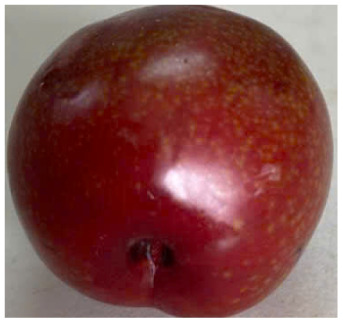	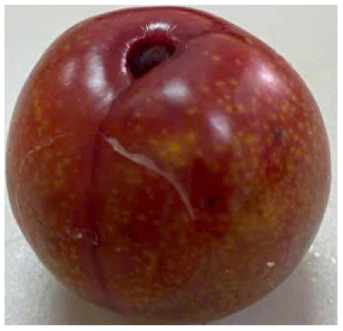
	CH1.5–GO2.0	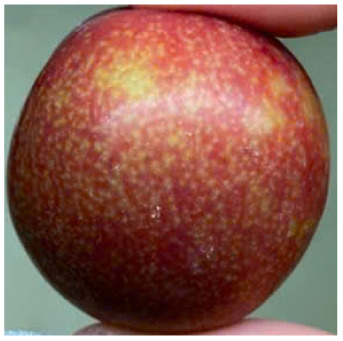	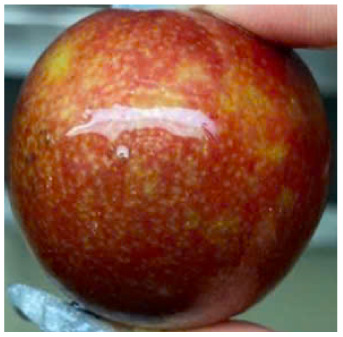	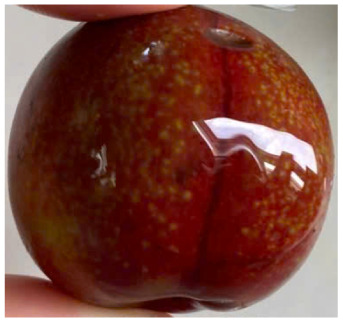	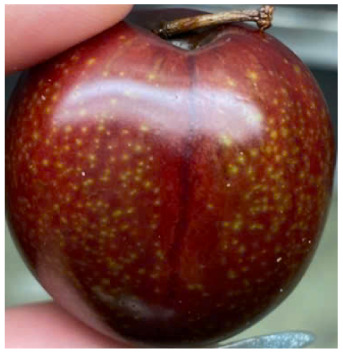	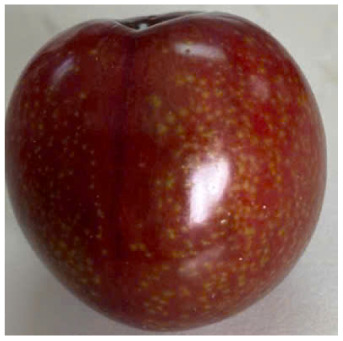	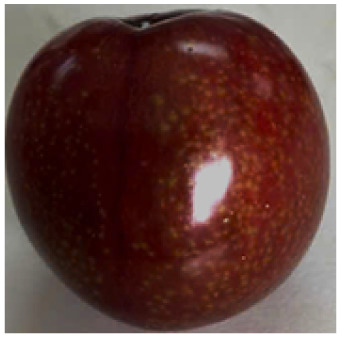

Most fruits showed slight stem-end spoilage by day 10, except those preserved in CH1.25–GO2.0, CH1.5–GO1.5, and CH1.5–GO2.0 films. Overall, uncoated plums began to spoil after 4–6 days. Preservation using a 1.25% CH film did not significantly improve shelf life, whereas the 1.5% CH film extended it by up to 8 days. Notably, films containing GO helped the fruits retain a brighter color, unaffected by the dark hue of GO, and maintained gloss for up to 10 days. By day 10, the fruits preserved in films with higher GO content (CH1.25–GO2.0, CH1.5–GO1.5, and CH1.5–GO2.0) remained undamaged, with firm, glossy skins and fresh appearance.

Visual observation results indicated that CH–GO films provided better preservation performance compared to cassava starch-based coatings, which maintained fruit quality for only about 8 days.^3^

**Fig. 7:**
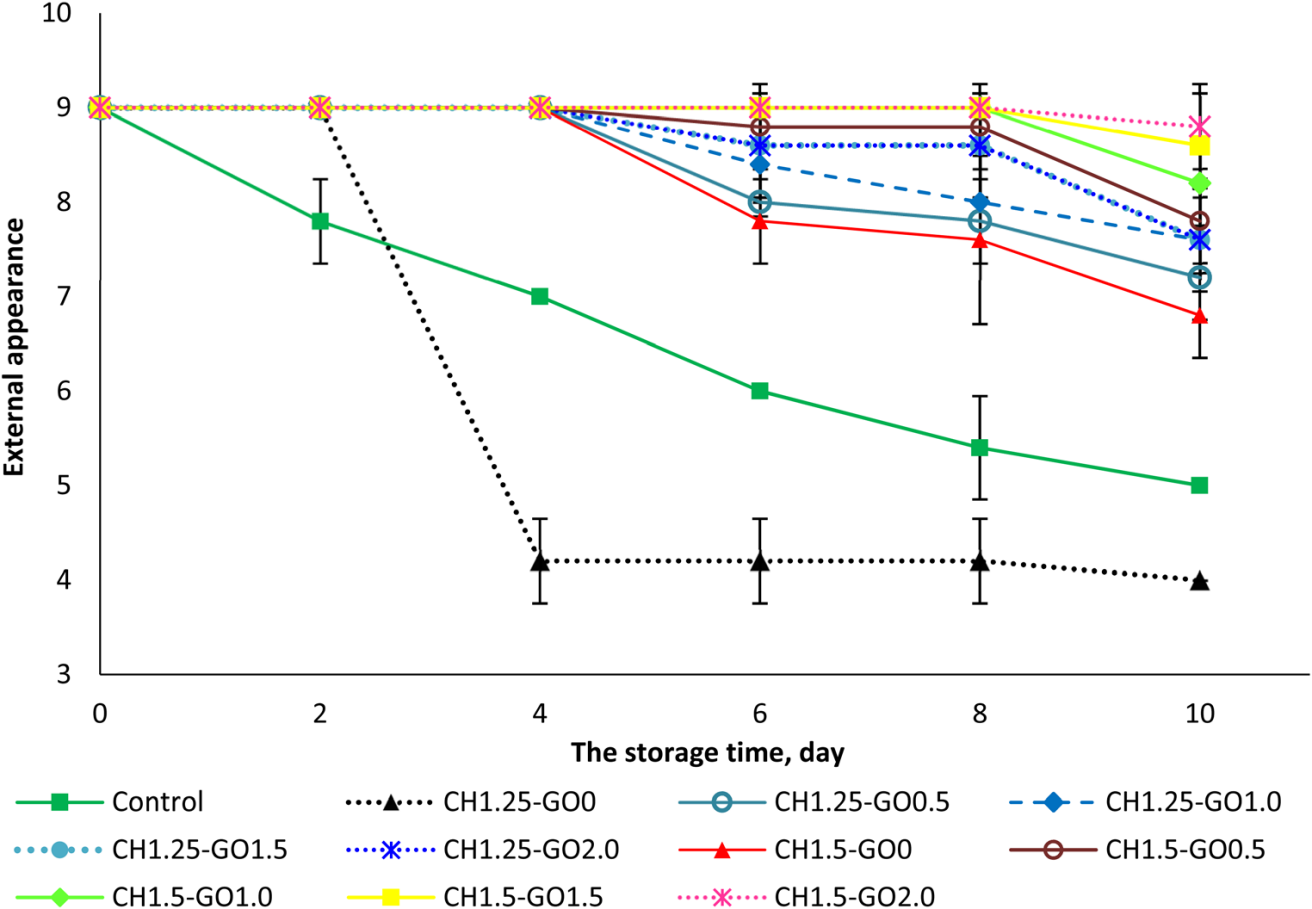
Evaluation of the external surface of plums during storage using the studied films over the storage period.

The evaluation of the external appearance of the fruit groups is presented in Fig. 7. Over the storage period, the external appearance scores of all samples gradually decreased; however, there were apparent differences among the treatment groups. Specifically, in the control group, the external appearance score declined most rapidly, starting to decrease on the second day of storage and reaching only 5 after 10 days, indicating severe surface wrinkling and loss of gloss.

For the group coated with the CH film at a CH concentration of 1.25%, the perfect appearance score (9 points) was maintained until the second day. Still, a sharp decrease was observed from the fourth day onward, due to visible surface deterioration, resulting in a score below the acceptable quality level. In contrast, the fruit groups coated with CH films containing GO and those with 1.5% CH maintained a score of 9 up to the fourth day, which then slightly decreased to 7.2–8.8 by the tenth day of storage, suggesting that the fruits were still rated as very good even after 10 days.

Notably, the fruits coated with CH 1.5% films containing 1.5–2.0% GO maintained a perfect appearance until day 8, after which a slight decline in score was observed on day 10. These findings indicate that incorporating GO into CH films significantly improves the external appearance of the fruits during storage. This can be explained by the fact that the polymer network structure of the film becomes more compact and exhibits enhanced moisture barrier properties due to the presence of GO, thereby reducing water evaporation and minimizing surface wrinkling or shriveling.

In addition, GO contributes to the antibacterial performance of the film, slowing down the growth of microorganisms responsible for surface decay, thus helping to maintain the natural gloss and color of the fruits. These results are consistent with previous studies showing that adding GO to CH films enhances their mechanical properties, reduces water vapor permeability, and extends the storage life of fresh fruits.

Therefore, CH–GO films with GO contents of 1.0–1.5% exhibit relatively high preservation efficiency, maintaining the visual quality and freshness of plums for a longer storage period than pure CH films or the uncoated control group.

#### 3.10.2. Weight loss

The weight loss of the control fruits and preserved using CH–GO film coatings was presented in Table 6. The results indicated that weight loss increased with storage duration across all samples, including the control and those coated with various films. Notably, fruits coated with a 1.25% CH film exhibited the highest weight loss after 10 days of storage, even higher than the control sample. The low-concentration CH film was more susceptible to dissolution under storage conditions. As the fruit respires, the generated moisture can partially dissolve the CH layer on the fruit surface, leading to an uneven film structure. This may result in localized heat accumulation on the surface, which accelerates spoilage and decay, thereby significantly increasing weight loss. In contrast, fruits preserved with CH films incorporated with GO, especially at GO contents of 1.0% or higher, were able to maintain a relatively stable weight over the 10 day storage period. By the 10th day, compared with the control sample, the fruits preserved with CH–GO films reduced the mass loss by about 21.60–72.53% compared with the fruit samples preserved in films without GO and 16.10–42.63% compared with the control sample.

**Table 6:** Weight loss of fruits during the storage period^a^

Sample	Weight loss of fruits during the storage period, %				
	Day 2	Day 4	Day 6	Day 8	Day 10
Control	7.40 ± 0.20^bD^	11.85 ± 1.54^aC^	14.08 ± 0.55^bC^	17.93 ± 1.16^bB^	23.48 ± 1.85^bA^
CH1.25–GO0	11.59 ± 0.13^aE^	21.75 ± 0.56^bD^	24.64 ± 0.20^aC^	27.68 ± 0.40^aB^	30.80 ± 0.35^aA^
CH1.25–GO0.5	4.41 ± 0.97^cD^	11.92 ± 1.44^bC^	13.99 ± 1.42^bBC^	16.99 ± 1.29^bAB^	19.70 ± 0.44^cA^
CH1.25–GO1.0	0.43 ± 0.05^dB^	1.68 ± 0.72^cB^	3.51 ± 0.60^dB^	8.22 ± 1.48^cA^	11.06 ± 0.76^deA^
CH1.25–GO1.5	0.28 ± 0.05^dC^	0.99 ± 0.20^cC^	3.08 ± 0.13^dBC^	5.24 ± 0.97^fB^	8.75 ± 1.27^fgA^
CH1.25–GO2.0	0.74 ± 0.17^dB^	1.34 ± 0.35^cB^	5.60 ± 2.21^cA^	7.53 ± 0.31^cdA^	8.46 ± 0.50^fgA^
CH1.5–GO0	2.13 ± 0.08^dE^	3.51 ± 0.12^cD^	5.75 ± 0.48^cdC^	8.58 ± 0.36^cdefB^	13.47 ± 0.11^dA^
CH1.5–GO0.5	0.49 ± 0.05^dD^	1.08 ± 0.41^cCD^	3.32 ± 0.44^dBC^	5.20 ± 1.12^efB^	10.56 ± 0.69^defA^
CH1.5–GO1.0	0.68 ± 0.11^dD^	1.53 ± 0.04^cCD^	2.81 ± 0.81^dC^	6.26 ± 0.36^defB^	10.38 ± 0.69^defA^
CH1.5–GO1.5	0.40 ± 0.08^dC^	1.40 ± 0.32^cBC^	3.14 ± 0.59^dB^	7.34 ± 0.43^cdeA^	8.96 ± 1.09^efgA^
CH1.5–GO2.0	0.52 ± 0.19^dC^	1.80 ± 0.49^cBC^	3.23 ± 0.26^dB^	5.83 ± 0.75^defA^	7.26 ± 0.03^gA^

***P*-value in ANOVA 2-way**					
CH	0.000	0.000	0.000	0.000	0.000
GO	0.000	0.000	0.000	0.000	0.000
CH*GO	0.000	0.000	0.000	0.000	0.000

a Uppercase letters: one-way ANOVA analyzed by row; lowercase letters: two-way ANOVA analyzed by column.

During storage, fruits continue to respire and lose moisture. For the uncoated control samples, respiration and water loss occur more readily due to unrestricted exposure to environmental conditions, resulting in rapid weight reduction. CH films, which possess relatively high water absorption capacity (Fig. 6), can absorb moisture around the fruit surface, thereby accelerating dehydration. As a result, fruits coated with pure CH films (without GO) experienced a more significant weight loss. Using CH at higher concentrations enhanced the antibacterial properties of the film material itself, delaying spoilage and maintaining better fruit quality. Additionally, CH–GO composite films, with their porous and rough surface structure and dense GO distribution, provide a greater surface area for retaining moisture produced during respiration. This moisture became trapped within the interstitial spaces between GO particles, slowing down its diffusion to the surrounding environment. Consequently, a localized humid microenvironment is maintained around the fruit, preserving its freshness and reducing weight loss.^3^

The results of the two-way ANOVA (Table 6) showed that both factors, CH content and GO content, had statistically significant effects (*p* < 0.05) on fruit weight loss during storage, and the interaction effect (CH × GO) was also important at all time points. This indicates that the effectiveness of the films in reducing weight loss depends not only on the individual components but also on the structural interaction between CH and GO within the film matrix. The results obtained in this study are consistent with a previous report on the effectiveness of CH–GO films in reducing the weight loss of passion fruit during storage.^21^ Specifically, the incorporation of GO into the CH matrix enhanced the mechanical properties and decreased the water vapor permeability of the film, thereby limiting moisture evaporation and maintaining the fruit's weight throughout the storage period. This suggests that the moisture-control mechanism of CH–GO films could be broadly applicable to various high-moisture fruits, including plums, as demonstrated in the present study.

#### 3.10.3. Color difference

The color difference of the fruit during the storage period was recorded and presented in Table 7. The results showed that storage led to a significant increase in color variation, which was associated with the natural ripening process and browning caused by fruit dehydration. However, the extent of color change differed markedly among the control sample, the fruits coated with pure CH film, and those preserved with CH–GO films. Specifically, the control fruits exhibited the fastest rate of color change, indicating earlier ripening or spoilage. In contrast, fruits coated with pure CH films showed slower color changes than the control, but the changes were still more pronounced than those observed in fruits preserved with CH–GO films. Most fruits stored using CH–GO films -especially those with GO contents of 1.5% or higher – demonstrated a significant reduction in the rate of color change. These findings were consistent with previous reports on the effectiveness of CH–GO films in preserving initial color and delaying ripening in passion fruits.^21^

**Table 7:** Color differences of fruits during the storage period

Sample	Color differences of fruits during the storage period				
	Day 2	Day 4	Day 6	Day 8	Day 10
Control	7.23 ± 0.06^aD^	7.70 ± 0.17^aD^	8.50 ± 0.10^aC^	10.37 ± 0.35^aB^	12.07 ± 0.12^aA^
CH1.25–GO0	4.27 ± 0.31^bD^	4.50 ± 0.17^bD^	5.13 ± 0.12^bC^	9.67 ± 0.15^aB^	10.60 ± 0.27^bcA^
CH1.25–GO0.5	3.07 ± 0.71^bcD^	4.77 ± 0.61^bC^	6.27 ± 0.47^bB^	7.30 ± 0.17^bAB^	8.13 ± 0.21^dA^
CH1.25–GO1.0	2.30 ± 0.27^cdeC^	2.60 ± 0.56^cdC^	6.27 ± 0.61^bB^	7.13 ± 0.47^bAB^	7.57 ± 0.25^dA^
CH1.25–GO1.5	1.30 ± 0.82^efC^	2.63 ± 0.61^cC^	5.00 ± 0.30^bB^	5.50 ± 0.20^cB^	7.10 ± 0.27^deA^
CH1.25–GO2.0	1.40 ± 0.56^defA^	1.40 ± 0.56^dA^	2.13 ± 0.60^cA^	2.63 ± 0.45^eA^	3.00 ± 0.85^gA^
CH1.5–GO0	3.87 ± 0.40^bD^	5.37 ± 0.31^bC^	5.87 ± 0.80^bC^	9.93 ± 0.38^aB^	11.67 ± 0.29^abA^
CH1.5–GO0.5	4.23 ± 0.15^bD^	5.47 ± 0.21^bC^	5.87 ± 0.23^bC^	7.90 ± 0.78^bB^	9.67 ± 0.42^cA^
CH1.5–GO1.0	2.50 ± 0.20^cdD^	5.40 ± 0.20^bC^	6.13 ± 0.55^bBC^	7.13 ± 0.40^bAB^	8.00 ± 0.52^dA^
CH1.5–GO1.5	1.97 ± 0.68^efC^	2.60 ± 0.27^cdC^	3.20 ± 0.56^cBC^	4.13 ± 0.21^dB^	5.40 ± 0.46^fA^
CH1.5–GO2.0	1.37 ± 0.67^fB^	1.70 ± 0.44^cdB^	2.40 ± 0.46^cB^	5.03 ± 0.57^cdA^	5.73 ± 0.31^efA^

***P*-value in ANOVA 2-way**					
CH	0.511	0.000	0.108	0.006	0.000
GO	0.000	0.000	0.000	0.000	0.000
CH*GO	0.088	0.000	0.004	0.000	0.000

During storage, respiration and physiological ripening were the primary factors contributing to color changes. A slower rate of color change suggests that the respiration and ripening processes were suppressed. Under normal conditions, fruits were exposed to high levels of environmental oxygen, which accelerates respiration and, consequently, the ripening process, leading to rapid color changes. In the case of fruits coated with pure CH films, the relatively smooth film surface acted as a barrier, limiting oxygen contact with the fruit and partially reducing respiration. However, the flat surface of the film could also absorb and retain heat, potentially promoting spoilage and accelerating color change. On the other hand, CH–GO films had a rougher surface with a dense distribution of GO particles, which increased the surface area available for trapping CO_2_ – one of the by-products of respiration. The accumulated CO_2_ surrounding the fruit created a high-CO_2_ microenvironment, which helped inhibit respiration and thereby slowed down the ripening and degradation processes. As a result, the fruit's color was better maintained over an extended period.

Two-way ANOVA showed that CH and GO content, and their interaction all had statistically significant effects (*p* < 0.05) on plum color change during storage, demonstrating the synergistic effect of these two factors in controlling the rate of color alteration and extending plum freshness. Therefore, the combination of CH and GO can be considered a promising biobased packaging approach for anthocyanin-rich fruits such as plums, helping to extend storage life, preserve natural color, and minimize postharvest losses.

#### 3.10.4. Total soluble solids (TSS)

The total soluble solids (TSS) content of the control fruits and preserved film was presented in Table 8. The results showed that, after 10 days of storage, the TSS content in the control samples as well as in the fruits preserved with CH1.25–GO0 and CH1.25–GO0.5 films significantly decreased. This indicated that a portion of the soluble solids was lost during the storage period. In contrast, the fruits preserved with GO-containing films maintained relatively stable TSS values, with negligible reductions compared to the initial TSS. The changes in TSS during storage were mainly attributed to the fruit's respiratory activity or decomposition when the fruit started to spoil or rot.^1^ The stable TSS levels observed in the fruits preserved with CH–GO films suggested that GO could slow down ripening and respiration processes, thereby extending freshness and delaying deterioration compared to the control samples. A similar positive effect on TSS was also reported when plums were coated with edible films based on CMC and pectin,^1^ and when passion fruit was preserved using CH–GO films.^21^ However, this finding contrasts with the earlier report by Vilvert, in which TSS increased during the storage of mangoes coated with CH–GO films.^30^ This discrepancy may stem from the differences in physiological characteristics and tissue structure among fruit types. Plums and passion fruits, which have higher water content and softer tissues, are more prone to water loss and rapid sugar degradation. In contrast, mangoes possess denser tissue structures and lower respiration rates, leading to a gradual accumulation of soluble sugars during postharvest ripening. Furthermore, variations in chemical composition, enzyme activity related to sugar metabolism, and environmental conditions (*e.g.*, temperature, humidity, and storage duration) may also contribute to the different TSS trends observed across studies.

**Table 8:** TSS of fruits during the storage period

Sample	TSS of fruits during the storage period, °Bx					
	Day 0	Day 2	Day 4	Day 6	Day 8	Day 10
Control	12.25 ± 0.25^aA^	12.10 ± 0.10 ^aA^	12.05 ± 0.15^abcA^	11.90 ± 0.10^abAB^	11.45 ± 0.05^bBC^	11.10 ± 0.10^cdC^
CH1.25–GO0	12.25 ± 0.25^aA^	12.30 ± 0.30^aA^	11.83 ± 0.15^bcAB^	11.47 ± 0.15^bABC^	11.20 ± 0.10^bBC^	10.80 ± 0.46^dC^
CH1.25–GO0.5	12.25 ± 0.25^aA^	12.07 ± 0.21 ^aA^	11.77 ± 0.15^cAB^	11.68 ± 0.13^abAB^	11.27 ± 0.25^bB^	11.37 ± 0.12^bcdB^
CH1.25–GO1.0	12.25 ± 0.25^aAB^	12.30 ± 0.27^aA^	12.30 ± 0.10^abA^	12.23 ± 0.21^aAB^	12.27 ± 0.06^aA^	11.63 ± 0.06^abcB^
CH1.25–GO1.5	12.25 ± 0.25^aA^	12.27 ± 0.46^aA^	12.37 ± 0.06^aA^	12.37 ± 0.15^aA^	12.33 ± 0.15^aA^	12.07 ± 0.21^aA^
CH1.25–GO2.0	12.25 ± 0.25^aA^	12.13 ± 0.12^aA^	12.20 ± 0.17^abcA^	12.07 ± 0.06^abA^	12.10 ± 0.46^aA^	12.00 ± 0.10^abA^
CH1.5–GO0	12.25 ± 0.25^aA^	12.20 ± 0.17^aA^	12.13 ± 0.23^abcA^	12.07 ± 0.06^abA^	12.33 ± 0.06^aA^	12.07 ± 0.25^aA^
CH1.5–GO0.5	12.25 ± 0.25^aA^	12.33 ± 0.12^aA^	12.23 ± 0.31^abcA^	12.03 ± 0.55^abA^	12.13 ± 0.15^aA^	12.03 ± 0.25^aA^
CH1.5–GO1.0	12.25 ± 0.25^aA^	12.27 ± 0.21^aA^	12.30 ± 0.17^abA^	12.13 ± 0.15^abA^	12.13 ± 0.12^aA^	12.27 ± 0.15^aA^
CH1.5–GO1.5	12.25 ± 0.25^aA^	12.17 ± 0.60^aA^	12.20 ± 0.10^abcA^	12.27 ± 0.15^aA^	12.23 ± 0.06^aA^	12.17 ± 0.29^aA^
CH1.5–GO2.0	12.25 ± 0.25^aA^	12.30 ± 0.40^aA^	12.17 ± 0.15^abcA^	12.27 ± 0.45^aA^	12.27 ± 0.21^aA^	12.13 ± 0.21^aA^

***P*-value in ANOVA 2-way**						
CH	0.000	0.738	0.088	0.057	0.000	0.000
GO	0.000	0.992	0.009	0.006	0.000	0.000
CH*GO	0.000	0.804	0.028	0.127	0.000	0.002

The two-way ANOVA analysis revealed that both CH and GO concentrations, as well as their interaction (CH × GO), had statistically significant effects (*p* < 0.05) on changes in total soluble solids during storage. This result indicates that the synergistic combination of CH and GO plays a crucial role in maintaining the soluble solid content of the fruit, thereby helping to preserve its sensory quality and nutritional value over time.

#### 3.10.5. pH and titratable acid ity

The pH values and titratable acidity of control fruits and those preserved with the studied films throughout the storage period were presented in Tables 9 and 10. The data indicated that the pH of all fruit samples tended to increase slightly or remain stable over the 10 day storage period. Specifically, the pH of the control fruits, fruits preserved with CH films without GO, and fruits preserved with the CH1.25–GO0.5 film showed an upward trend over time. In contrast, the pH of the remaining samples exhibited only slight and insignificant change, indicating a relatively stable pH. This increase in pH was accompanied by a decrease in the total titratable acidity of fruits (Table 10). During postharvest storage, fruits continue to respire, a process that consumes certain nutrients, including organic acids such as malic acid, which plays a direct role in the respiration cycle.^1^ When respiration was intense, the total acidity of the fruit decreased, resulting in a rise in pH. Additionally, the use of organic acids as carbon skeletons for synthesizing new compounds within the fruit may further contribute to the reduction in acid content.

**Table 9:** pH of control fruits and fruits preserved with the studied films during the storage period

Sample	pH of fruits during the storage period					
	Day 0	Day 2	Day 4	Day 6	Day 8	Day 10
Control	3.19 ± 0.05^aC^	3.24 ± 0.03^aBC^	3.26 ± 0.03^aABC^	3.27 ± 0.09^aABC^	3.39 ± 0.07^aAB^	3.44 ± 0.13^abA^
CH1.25–GO0	3.19 ± 0.05^aB^	3.20 ± 0.13^aB^	3.25 ± 0.05^aB^	3.27 ± 0.07^aAB^	3.35 ± 0.06^abAB^	3.47 ± 0.07^aA^
CH1.25–GO0.5	3.19 ± 0.05^aB^	3.22 ± 0.03^aB^	3.25 ± 0.02^aAB^	3.27 ± 0.02^aAB^	3.31 ± 0.05^abA^	3.32 ± 0.02^abcA^
CH1.25–GO1.0	3.19 ± 0.05^aA^	3.24 ± 0.02^aA^	3.24 ± 0.01^aA^	3.24 ± 0.01^aA^	3.26 ± 0.02^bA^	3.25 ± 0.03^cA^
CH1.25–GO1.5	3.19 ± 0.05^aA^	3.22 ± 0.06^aA^	3.26 ± 0.07^aA^	3.22 ± 0.04^aA^	3.23 ± 0.04^bA^	3.26 ± 0.03^cA^
CH1.25–GO2.0	3.19 ± 0.05^aA^	3.21 ± 0.04^aA^	3.24 ± 0.03^aA^	3.24 ± 0.02^aA^	3.23 ± 0.04^bA^	3.24 ± 0.04^cA^
CH1.5–GO0	3.19 ± 0.05^aB^	3.25 ± 0.02^aAB^	3.26 ± 0.01^aAB^	3.27 ± 0.01^aA^	3.29 ± 0.04^abA^	3.30 ± 0.03^bcA^
CH1.5–GO0.5	3.19 ± 0.05^aA^	3.21 ± 0.03^aA^	3.22 ± 0.06^aA^	3.22 ± 0.04^aA^	3.23 ± 0.05^bA^	3.24 ± 0.01^cA^
CH1.5–GO1.0	3.19 ± 0.05^aA^	3.21 ± 0.03^aA^	3.23 ± 0.04^aA^	3.23 ± 0.05^aA^	3.24 ± 0.03^bA^	3.26 ± 0.05^cA^
CH1.5–GO1.5	3.19 ± 0.05^aA^	3.22 ± 0.04^aA^	3.22 ± 0.07^aA^	3.23 ± 0.02^aA^	3.24 ± 0.02^bA^	3.26 ± 0.03^cA^
CH1.5–GO2.0	3.19 ± 0.05^aA^	3.23 ± 0.03^aA^	3.25 ± 0.02^aA^	3.25 ± 0.03^aA^	3.24 ± 0.06^bA^	3.24 ± 0.07^cA^

***P*-value in ANOVA 2-way**						
CH	0.000	0.395	0.604	0.785	0.368	0.083
GO	0.000	0.824	0.959	0.899	0.297	0.004
CH*GO	0.000	0.975	0.955	0.987	0.396	0.029

**Table 10:** Titratable acidity of control fruits and fruits preserved with the studied films during the storage period

Sample	Titratable acidity of the fruit during the storage period mqE l^−1^					
	Day 0	Day 2	Day 4	Day 6	Day 8	Day 10
Control	1.89 ± 0.03^aA^	1.85 ± 0.05^aA^	1.81 ± 0.06^aA^	1.76 ± 0.10^aA^	1.57 ± 0.11^aAB^	1.42 ± 0.13^bB^
CH1.25–GO0	1.89 ± 0.03^aA^	1.84 ± 0.05^aAB^	1.80 ± 0.07^aAB^	1.75 ± 0.11^aAB^	1.62 ± 0.11^aBC^	1.43 ± 0.10^bC^
CH1.25–GO0.5	1.89 ± 0.03^aA^	1.87 ± 0.04^aA^	1.84 ± 0.06^aA^	1.79 ± 0.10^aAB^	1.65 ± 0.06^aBC^	1.57 ± 0.07^abC^
CH1.25–GO1.0	1.89 ± 0.03^aA^	1.86 ± 0.05^aA^	1.83 ± 0.07^aA^	1.79 ± 0.10^aA^	1.78 ± 0.08^aA^	1.78 ± 0.08^aA^
CH1.25–GO1.5	1.89 ± 0.03^aA^	1.85 ± 0.03^aA^	1.81 ± 0.07^aA^	1.79 ± 0.07^aA^	1.77 ± 0.07^aA^	1.73 ± 0.08^aA^
CH1.25–GO2.0	1.89 ± 0.03^aA^	1.85 ± 0.03^aA^	1.81 ± 0.05^aA^	1.78 ± 0.08^aA^	1.76 ± 0.08^aA^	1.73 ± 0.08^aA^
CH1.5–GO0	1.89 ± 0.03^aA^	1.86 ± 0.05^aAB^	1.82 ± 0.07^aAB^	1.77 ± 0.10^aAB^	1.71 ± 0.14^aAB^	1.65 ± 0.08^abB^
CH1.5–GO0.5	1.89 ± 0.03^aA^	1.88 ± 0.03^aA^	1.83 ± 0.05^aAB^	1.81 ± 0.05^aAB^	1.78 ± 0.05^aAB^	1.75 ± 0.04^abB^
CH1.5–GO1.0	1.89 ± 0.03^aA^	1.81 ± 0.05^aAB^	1.81 ± 0.04^aAB^	1.80 ± 0.03^aAB^	1.80 ± 0.05^aAB^	1.78 ± 0.04^aB^
CH1.5–GO1.5	1.89 ± 0.03^aA^	1.88 ± 0.03^aA^	1.85 ± 0.03^aA^	1.82 ± 0.05^aA^	1.78 ± 0.08^aA^	1.73 ± 010^aA^
CH1.5–GO2.0	1.89 ± 0.03^aA^	1.86 ± 0.04^aA^	1.82 ± 0.06^aA^	1.79 ± 0.08^aA^	1.74 ± 0.11^aA^	1.71 ± 0.11^aA^

***P*-value in ANOVA 2-way**						
CH	0.000	0.751	0.499	0.555	0.089	0.005
GO	0.000	0.996	0.881	0.290	0.012	0.000
CH*GO	0.000	0.775	0.867	0.702	0.289	0.004

Notably, fruits preserved with CH films containing GO exhibited remarkable stability in both pH and titratable acidity. This can be attributed to the layered and porous structure of GO, which enables it to retain CO_2_ released during fruit respiration. The accumulation of CO_2_ around the fruit creates a CO_2_-rich environment, which suppresses the respiration rate and thereby slows the degradation of organic acids, helping to maintain a stable pH.^51^

Similar observations regarding the reduction of acid content in fruits have also been reported when plums were preserved using alginate coating films,^52^ as well as edible coatings made from CMC, pectin, or combinations of pectin and CMC.^1^ These findings suggest that the application of polysaccharide-based coatings can effectively slow down acid degradation, likely by forming a semi-permeable barrier that reduces gas exchange and moisture loss, thereby limiting metabolic processes such as respiration and organic acid breakdown. Moreover, the consistency of these results across different coating materials highlights the broader potential of biopolymer-based films to maintain fruit quality, not only by preserving acidity but also by contributing to overall shelf-life extension and sensory attributes. This provides a rationale for further exploring composite coatings, such as CH–GO films, which may combine mechanical reinforcement and barrier properties with additional functional benefits like antimicrobial activity. For plums – fruits characterized by high water content, thin skin, and high perishability – the use of packaging materials capable of preventing moisture loss is essential to maintain freshness. In addition, packaging with good mechanical strength is crucial to minimize impact and mechanical damage during transportation, storage, and distribution, thereby preserving fruit quality. Moreover, the antibacterial activity of the packaging material helps inhibit the growth of spoilage microorganisms, preventing microbial degradation processes that can deteriorate fruit quality. Particularly for fruits rich in anthocyanins and antioxidant compounds, such as plums, the UV-shielding ability of the packaging film is also a key factor in maintaining freshness, color, and nutritional value throughout storage. From these perspectives, the CH–GO film exhibits great potential for packaging and preserving thin-skinned, highly respiring fruits like plums, where maintaining moisture and minimizing water loss are crucial to protect freshness, juiciness, and natural color. Furthermore, the optical properties of GO help reduce direct light exposure, indirectly protecting light-sensitive pigments such as anthocyanins, thereby extending shelf life and enhancing the commercial value of the fruits.

The application of CH–GO films in food preservation not only helps maintain the postharvest quality of fruits but also offers significant economic and environmental benefits. Owing to their ability to extend shelf life and reduce weight loss by up to 72%, these films can substantially minimize postharvest losses, which account for a significant portion of the total cost in the fruit supply chain. Reducing spoilage not only helps prevent resource waste but also provides consumers with fresh, high-quality products at a reasonable cost. Therefore, the use of biodegradable preservation films in general, and CH–GO composite films in particular, can be considered an economical and efficient packaging solution to address postharvest loss issues. Moreover, compared with other preservation films containing metal nanoparticles, CH–GO films exhibit significantly lower production costs while still maintaining excellent preservation performance. Importantly, replacing metal oxide–based materials such as silver oxide, zinc oxide, or titanium oxide with CH–GO films can help eliminate the risk of heavy metal residues in packaging and food products, thereby ensuring consumer safety and promoting environmental sustainability.

From an environmental perspective, the CH–GO film is entirely composed of naturally derived materials with high biodegradability. The combination of CH and GO not only makes effective use of renewable resources obtained from agricultural and seafood by-products such as shrimp and crab shells, but also contributes to the development of a new, environmentally friendly packaging material. This film can serve as a sustainable alternative to conventional plastic packaging, helping to reduce non-biodegradable solid waste and lessen dependence on petroleum-based materials. Owing to its rapid biodegradability, the use of CH–GO film significantly contributes to mitigating the accumulation of solid waste in the environment. With these advantages, the CH–GO film not only functions as an efficient packaging material for food preservation but also aligns well with the global trends toward sustainable development and the circular economy.

Although this study clearly demonstrates the potential of CH–GO films for food preservation in general, and for dark-colored fresh fruits such as plums, grapes, and black strawberries in particular, certain inherent limitations exist in the experimental design.

(1) Limited sample size: the preservation experiments were conducted using five fruits per group, primarily to ensure uniformity in ripeness and quality during the testing period. While this sample size limits the representativeness, it helps minimize variability and provides valuable preliminary data on the effects of CH–GO films on fruit quality. Expanding the sample size in future studies could enhance the statistical reliability and validate the results on a larger scale.

(2) Specific storage conditions: the study focused on evaluating the effects of different chitosan-to-GO ratios under ambient conditions over a short duration. This approach allows for a clearer understanding of the interaction mechanisms between the film and the fruit before extending the investigation to other storage conditions, such as cold storage or combinations with practical preservation methods.

(3) Microbiological assessment not included: microbial indicators on the fruit surface were not analyzed in the current study. Nevertheless, the observed antimicrobial properties of CH–GO films provide initial evidence of their potential to limit spoilage-causing microorganisms. Future microbiological analyses will further strengthen the understanding of the film's preservation capabilities.

(4) Limited fruit types: the study focused on plums to maintain uniformity and facilitate observation of preservation indicators. Testing other fruit types will be an essential step to broaden the applicability of CH–GO films.

(5) In addition, further research on the safety of chitosan (CH) and graphene oxide (GO) components when applied in preservative films is necessary for future studies. Evaluations of the viability, migration levels, and safety of GO and CH in direct contact with food will provide important evidence, supporting the expansion of the potential applications of CH–GO materials in food packaging systems at a practical scale.

These limitations primarily reflect the exploratory nature of this initial study and provide a reasonable basis for developing subsequent research on a larger scale and broader scope, ultimately guiding the practical application of CH–GO films in fresh fruit preservation.

## 4 Conclusion

This study investigated the characteristics of chitosan-based biopolymer films incorporated with varying concentrations of GO for potential application in packaging fresh plums. The results showed that the addition of GO at low concentrations helped maintain the transparency of the films. At the same time, higher GO contents significantly altered their morphology and color, resulting in darker and less vivid films compared to pure chitosan. The CH–GO films also exhibited improved UV-blocking ability and significantly reduced water absorption, solubility, and water vapor permeability. In terms of mechanical properties, films containing low levels of GO showed higher tensile strength, while the elongation at break improved only when GO was added at concentrations of 1.5% or higher. Although the antioxidant activity decreased with GO incorporation, the antibacterial performance – particularly against *E. coli* – was markedly enhanced. Thus, the incorporation of GO into CH matrices effectively improved several essential properties of the biopolymer films, including mechanical strength, water vapor barrier capacity, and antimicrobial activity. Moreover, CH–GO films exhibited relatively positive effects in maintaining the quality and extending the shelf life of fresh plums. These findings highlight the potential of CH–GO composite films as promising bio-based packaging materials for various fresh agricultural products that are sensitive and highly perishable. Thanks to its sustainability and potential to replace conventional plastic packaging, chitosan-based material containing GO is considered a promising solution for bio-based packaging with excellent antibacterial and UV-shielding properties. However, to broaden its practical applicability, future studies should be conducted on a wider range of fruits, under various storage conditions, with more extended preservation periods and larger sample scales, as well as the safety and toxicity of CH–GO material to comprehensively evaluate the effectiveness of this material in fresh produce preservation.

## Author contributions

Dr Tran Y Doan Trang: study design and manuscript writing. Biological tests and antioxidant tests were performed by Dr Ha Thi-Dung. Experimental work was conducted by Phan Khiet Suong. GO synthesis was carried out by Dr Duong Van Thiet. SEM and FTIR analysis were supported by Prof. Nguyen The Huu. Grammar and language editing were done by Dr Vu Phuong Lan. Chemicals and materials were prepared by Prof. Nguyen Quang Tung.

## Conflicts of interest

The authors declare no conflict of interest.

## Data Availability

All data generated or analyzed during this study are included in this published article.

## References

[cit1] Panahirad S., Naghshiband-Hassani R., Bergin S., Katam R., Mahna N. (2020). Plants.

[cit2] Roussos P. A., Efstathios N., Intidhar B., Denaxa N.-K., Tsafouros A. (2016). Nutritional Composition of Fruit Cultivars.

[cit3] Nogueira G. F., Leme B. de O., S dos Santos G. R. (2021). et al.. Polysaccharides.

[cit4] D. S., Sanjana S., Madu B., Nivetha K., Kandasamy A. (2019). Int. J. Innov. Res. Sci. Technol..

[cit5] Igwe E. O., Charlton K. E. (2016). Phytother Res..

[cit6] FAOSTAT , Plum Production by Country 2025, World Population Review, 2025

[cit7] Pillai A. R. S., Eapen A. S., Zhang W., Roy S. (2024). Foods.

[cit8] Motelica L., Ficai D., Ficai A., Oprea O. C., Kaya D. A., Andronescu E. (2020). Foods.

[cit9] Wrońska N., Anouar A., El Achaby M., Zawadzka K., Kędzierska M., Miłowska K., Katir N., Draoui K., Różalska S., Piwoński I. (2020). et al.. Materials.

[cit10] An N., Zhu X., Xu S., Lin X., Zhu Y., Huang M. Y., Xu Q., Xue F., Wu L. (2024). Chem. Eng. J..

[cit11] Mohanty F., Swain S. K. (2017). Nanotechnol. Appl. Food.

[cit12] Lou M.-M. (2011). et al.. Carbohydr. Res..

[cit13] Trang Y. D. T., Zenitova L. A. (2019). IOP Conf. Ser. Earth Environ. Sci..

[cit14] Sahariah P., Másson M. (2017). Biomacromolecules.

[cit15] Muthuchamy M. (2020). et al.. Carbohydr. Polym..

[cit16] Trang T. Y. D., Dzung H. T., Huong T. T., Dien L. Q., Hanh D. T., Phuong H. T. N. (2023). E3S Web Conf..

[cit17] Trang T. Y. D., Dzung H. T., Huong T. T., Quynh P. H. (2024). J. Phys.:Conf. Ser..

[cit18] Zabihi E., Babaei A., Shahrampour D., Arab-Bafrani Z., Mirshahidi K. S., Majidi H. J. (2019). Int. J. Biol. Macromol..

[cit19] Frindy S. (2017). et al.. Carbohydr. Polym..

[cit20] Hummers W. S., Offeman R. E. (1958). J. Am. Chem. Soc..

[cit21] da Silva W. A. O. (2024). et al.. Horticulturae.

[cit22] Szunerits S., Boukherroub R. (2016). J. Mater. Chem. B.

[cit23] Vilas C., Mauricio-Iglesias M., García M. R. (2020). Food Packag. Shelf Life.

[cit24] Valencia Zapata M. E. (2019). et al.. Int. J. Mol. Sci..

[cit25] Kosowska K., Domalik-Pyzik P., Krok-Borkowicz M., Chłopek J. (2019). Materials.

[cit26] Prakash J., Prema D., Venkataprasanna K. S., Balagangadharan K., Selvamurugan N., Venkatasubbu G. D. (2020). Int. J. Biol. Macromol..

[cit27] Sabzevari M., Cree D. E., Wilson L. D. (2018). ACS Omega.

[cit28] Han Lyn F., Tan C. P., Zawawi R. M., Nur Hanani Z. A. (2021). Food Hydrocoll..

[cit29] Tran N. T., Nguyen G. T., Le T. M., Huynh A. T. N. (2025). J. Appl. Polym. Sci..

[cit30] Vilvert J. C. (2022). et al.. LWT--Food Sci. Technol..

[cit31] Paiva C. A. (2020). et al.. J. Food Process. Preserv..

[cit32] Koosha M. (2021). et al.. J. Funct. Biomater..

[cit33] Durmus D. and Davis W., Proceedings of the 29^th^ Quadrennial Session of the CIE, International Commission on Illumination, CIE, 2019, pp. 888–895, 10.25039/x46.2019.PO005

[cit34] ASTM E96-00e1: Standard Test Methods for Water Vapor Transmission of Materials, American Society for Testing and Materials, West Conshohocken, PA, USA, 1989

[cit35] Trang T. Y. D., Dzung H. T., Lan V. P., Duc H. T., Hanh D. T. (2025). E3S Web Conf..

[cit36] Nogueira G. F., Fakhouri F. M., de Oliveira R. A. (2018). Carbohydr. Polym..

[cit37] Trang T. Y. D. (2024). et al.. Food Sci. Technol..

[cit38] Han D., Yan L., Chen W., Li W. (2011). Carbohydr. Polym..

[cit39] Gea S., Sari J. N., Bulan R., Piliang A., Amaturrahim S. A., Hutapea Y. A. (2018). J. Phys.:Conf. Ser..

[cit40] Emiru T. F., Ayele D. W. (2017). Egyptian Journal of Basic and Applied Sciences.

[cit41] Zuo P.-P. (2013). et al.. Chem. Cent. J..

[cit42] Ahmed J., Mulla M., Arfat Y. A., Thai T L. A. (2017). Food Hydrocoll..

[cit43] Boeva Z. A., Milakin K. A., Pesonen M., Ozerin A. N., Sergeyev V. G., Lindfors T. (2014). RSC Adv..

[cit44] Yang Y., Wang Q., Qiu W., Guo H., Gao F. (2016). J. Phys. Chem. C.

[cit45] Arfat Y. A., Ahmed J., Ejaz M., Mullah M. (2017). Int. J. Biol. Macromol..

[cit46] Jamróz E. (2016). et al.. Polymers.

[cit47] Aydoğan C. (2021). et al.. Electrophoresis.

[cit48] Chen Y., Pandit S., Rahimi S., Mijakovic I. (2021). Adv. Mater. Interfaces.

[cit49] Radhi A., Mohamad D., Abdul Rahman F. S., Abdullah A. M., Hasan H. (2021). J. Mater. Res. Technol..

[cit50] Pandit S., Gaska K., Kádár R., Mijakovic I. (2021). ChemPhysChem.

[cit51] Shi J., Tong R., Yao J., Wang S., Wang S., Li J., Song C., Zhang K., Jiao J., Wang M., Hao P., Zhao Y., Xu W., Liu Y., Wan R., Zheng X. (2025). Controlled atmosphere storage with high CO_2_ concentration extends storage life of fresh pomegranate fruit by regulating antioxidant capacity and respiration metabolism. BMC Plant Biol..

[cit52] Valero D. (2013). et al.. Postharvest Biol. Technol..

